# Natural killer cells and innate lymphoid cells 1 tune anxiety-like behavior and memory in mice via interferon-γ and acetylcholine

**DOI:** 10.1038/s41467-023-38899-3

**Published:** 2023-05-29

**Authors:** Stefano Garofalo, Germana Cocozza, Alessandro Mormino, Giovanni Bernardini, Eleonora Russo, Donald Ielpo, Diego Andolina, Rossella Ventura, Katiuscia Martinello, Massimiliano Renzi, Sergio Fucile, Mattia Laffranchi, Eva Piano Mortari, Rita Carsetti, Giuseppe Sciumè, Silvano Sozzani, Angela Santoni, Marie-Eve Tremblay, Richard M. Ransohoff, Cristina Limatola

**Affiliations:** 1 Department of Physiology and Pharmacology, Sapienza University of Rome, Rome, Italy; 2 IRCCS Neuromed, Pozzilli, Italy; 3 Department of Molecular Medicine, Sapienza University of Rome, Rome, Italy; 4 Department of Psychology and Centre for Research in Neurobiology D. Bovet, Sapienza University of Rome, Rome, Italy; 5 B Cell Unit, Immunology Research Area, Bambino Gesù Children’s Hospital, IRCCS, Rome, Italy; 6 Centre de Recherche CHU de Quebec-Université Laval, Quebec City, QC Canada; 7 Third Rock Ventures, Boston, MA USA; 8 Department of Physiology and Pharmacology, Sapienza University, Laboratory affiliated to Istituto Pasteur, Rome, Italy

**Keywords:** Neuroimmunology, Neurophysiology

## Abstract

The mechanisms of communication between the brain and the immune cells are still largely unclear. Here, we characterize the populations of resident natural killer (NK) cells and innate lymphoid cells (ILC) 1 in the meningeal dura layer of adult mice. We describe that ILC1/NK cell-derived interferon-γ and acetylcholine can contribute to the modulation of brain homeostatic functions, shaping synaptic neuronal transmission and neurotransmitter levels with effects on mice behavior. In detail, the interferon-γ plays a role in the formation of non-spatial memory, tuning the frequency of GABAergic neurotransmission on cortical pyramidal neurons, while the acetylcholine is a mediator involved in the modulation of brain circuitries that regulate anxiety-like behavior. These findings disclose mechanisms of immune-to-brain communication that modulate brain functions under physiological conditions.

## Abstract (web summary)

The meningeal compartment communicates with the brain to modulate homeostatic functions. Here, the authors demonstrate that natural killer (NK) cells and innate lymphoid cells (ILC) 1 shape synaptic neuronal transmission and affect mouse behavior.

## Introduction

The meningeal compartment hosts both innate and adaptive immune cells, providing constant immunosurveillance of the CNS in a highly dynamic microenvironment^1–11^. Bidirectional communication among the nervous and the immune system influences many physiological and pathological conditions^12,13^. Often, these neuroimmune interactions are mediated by soluble molecules that play distinct roles in the two systems, namely neurotransmitters and cytokines^12–15^.

Immune system activation in response to infectious agents or immune dysfunctions influences behavior^16,17^. It remains unclear whether and how immune cells participate in cognition and learning processes in physiological conditions. Meningeal immunity also participates in behavioral processes. In mice, T cells tune GABAergic neurotransmission through interleukin (IL)−4, −17 and interferon (IFN)-γ, and play crucial roles in social behavior, spatial learning and memory^8,18–23^.

Under physiological conditions, natural killer (NK) cells are barely detectable within the brain parenchyma, but they are present in the choroid plexus, and in the meningeal spaces^24,25^. In brain tumors and in different neuropathologies, NK cells enter the brain^26–29^, modify microglial phenotype^30–33^, and signal to neurons^34^. In aged mice and humans, NK cells increase in number in the hippocampal dentate gyrus, exerting toxic activity toward aged neuroblasts, with detrimental effects on synaptic plasticity and cognitive functions^35^.

Here, we characterized the meningeal NK cells and innate lymphoid cells (ILC) 1, and compare them with splenic cells. We also described that ILC1 and NK cells represent unexpected actors in the immune-mediated regulation of brain homeostasis, affecting neuronal activity, tuning specific neuronal networks and modulating mice behavior. We report that, in mice, the removal of NK1.1^+^ cells reduced the anxiety-like behavior and impaired the non-spatial memory potentially through IFN-γ and acetylcholine (ACh) release. The effects on anxiety-like behavior are associated with the modulation of hypothalamic-ventral tegmental area (VTA)-hippocampal network via a three steps mechanism: (1) activation of ACh-induced hypothalamic orexin neurons; (2) activation of dopaminergic neurons in the VTA and (3) dopaminergic stimulation of the hippocampal region. Memory impairment, differently, is mediated by IFN-γ which modulates the frequency of inhibitory spontaneous events on pyramidal cortical neurons.

Altogether, our findings reveal mechanisms of functional interactions among innate immunity and neurons under physiological conditions.

## Results

### Transcriptional landscape of meningeal-resident NK cells and ILC1

**Fig. 1:**
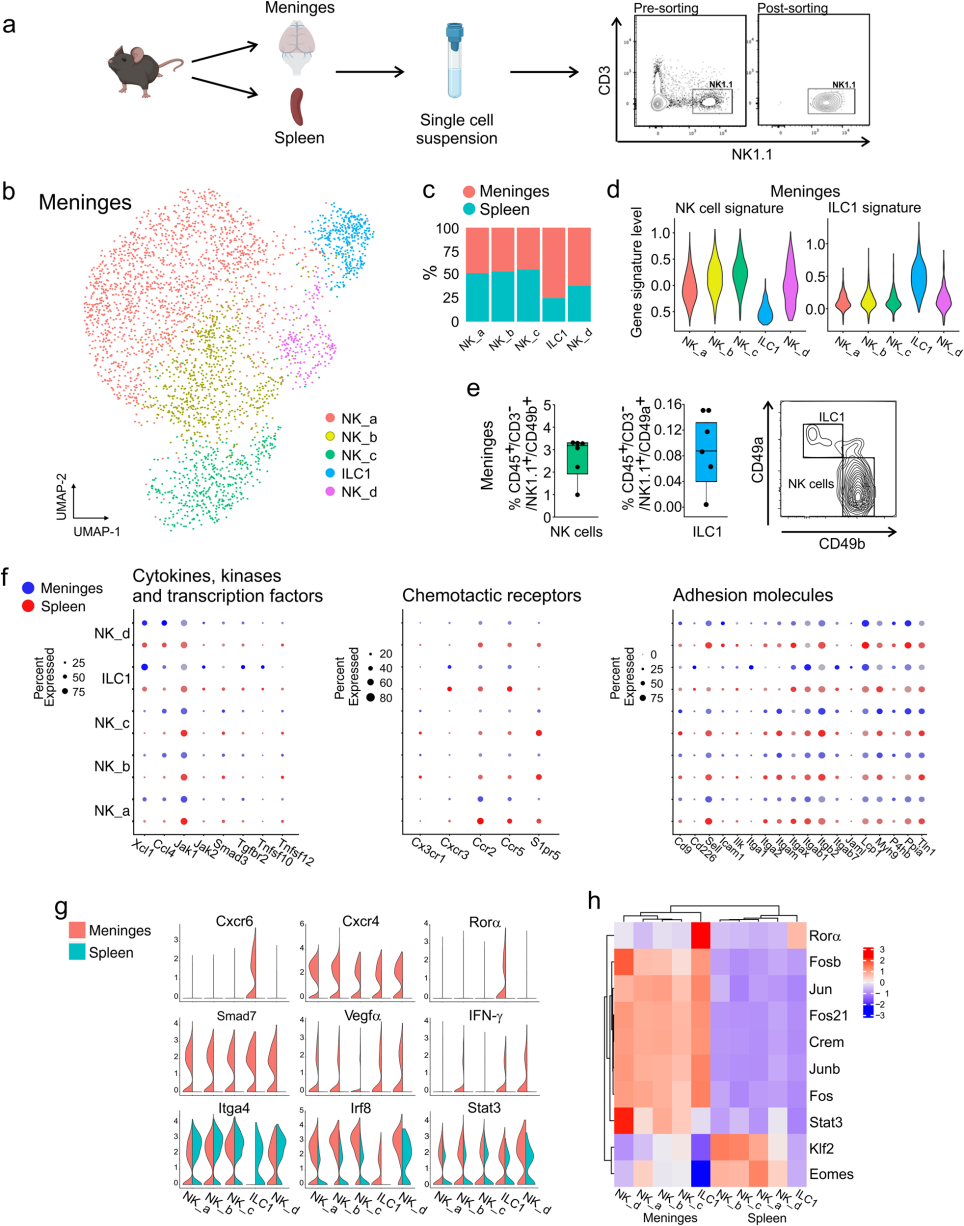
Transcriptional landscape of meningeal NK cells and ILC1. **a** Schematic overview of sorted meningeal or splenic CD3^−^/NK1.1^+^ cells. C57BL/6 mice (*n* = 50) were intracardially perfused with PBS, and CD3^−^/NK1.1^+^ cells were sorted from meninges or spleen. Figure created with BioRender.com. **b** UMAP representation of CD3^−^/NK1.1^+^ cells derived from meninges (3392 cells). Profiles colored are from clusters identified subsets of NK cells and ILC1. **c** Percentage of NK cell subsets and ILC1 obtained in the meninges and spleen. **d** Violin plots represent the distribution of ILC1 and NK cell gene expression programs defined in Robinette et al.^74^, grouped by clusters in the meninges. **e** NK cells and ILC1 frequency in the meninges of C57BL/6 mice (*n* = 6). Right: representative FACS analysis of meningeal ILC1/NK cells. For boxplots, the center line, boxes and whiskers represent the median, inner quartiles, and rest of the data distribution, respectively. **f** DotPlot represent the expression levels of ILCs archetypal genes within the identified NK cells and ILC1 clusters in meninges and spleen. **g** Violin plots represent the distribution of indicated genes grouped by clusters and divided for tissue. **h** Heatmap of the scaled regulon activity for select regulons expressed in each NK cell and ILC1 population.

To expand our understanding on the functions of individual meningeal cell populations, we investigated CD3^−^/NK1.1^+^ NK cells and ILC1, examining possible interactions with the brain. We first characterized these cell populations in adult male mice by single-cell RNA-sequencing (scRNA-seq) (Fig. 1a), identifying distinct NK cell clusters (a-d) and one ILC1 cluster (Fig. 1b–d; Supplementary Fig. S1a–c; Supplementary Table 1). The genes selected to determine the “NK cell signature” and “ILC1 signature” are reported in Supplementary Table 1. FACS analysis using CD49a and CD49b permitted to identify both NK cells and ILC1 in the meningeal compartment^24^ (Fig. 1e). Meningeal (dura layer) and splenic NK cells/ILC1 have unique expression patterns of chemokine receptors and adhesion molecules, suggesting the presence of different homing mechanisms. In the meninges, NK cells and ILC1 express *Cxcr4*, while *Cxcr6* is specifically expressed by ILC1. In the spleen, both express *Ccr2*, *Ccr5* and *S1pr5* (Fig. 1f, g; Supplementary Fig. S1d). Signaling through specific chemokine receptors could be involved in ILC1 and NK cells entry into the CNS upon damages^26,36^, but specific studies are necessary to address the potential role of brain-produced chemotactic signals. Further differences are observed in *Smad7, Vegfa* and *Ifn-g*, only expressed by meningeal ILC1/NK cells, and in transcription factors (TFs): meningeal NK cells are indeed enriched in *Irf8* and *Eomes*, while meningeal ILC1 express *Rora* (Fig. 1f, g). No differences were found in the expression of *Stat4*, required for optimal IFN-γ transcription^37^, and *Tbet* between splenic and meningeal ILC1/NK cells (Supplementary Fig. S1c), although FACS analysis revealed an increased STAT4 protein expression in meningeal NKp46^+^ cells in comparison to the splenic ones (Supplementary Fig. S1e). The tissue specific TF activity was supported by the SCENIC algorithm^38^ (Fig. 1h). We speculate that these differences could be due to different tissue origins^39^, to the innate lymphocyte plasticity^40^ or could highlight specific meningeal cell functions in comparison with peripheral cells^41^, worth of further studies.

### NK cells and ILC1 role in mice behavior

**Fig. 2:**
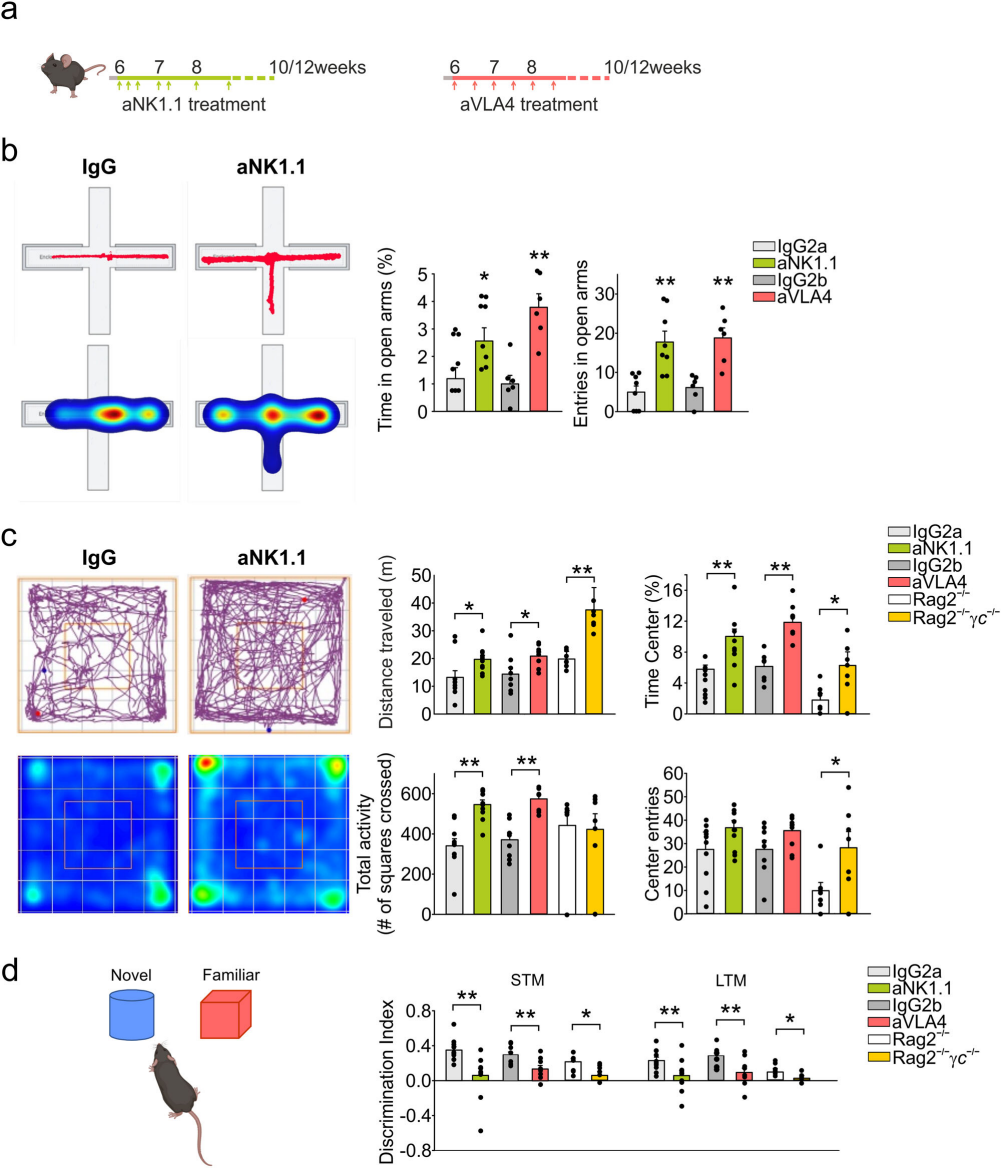
Role of NK cells and ILC1 in mice behavior. **a** Scheme of aNK1.1 and aVLA4 treatments in C57BL/6 mice. **b** The percentage of time spent and the frequency of entries in the open arms in elevated plus maze in IgG2a and aNK1.1 treated mice (*n* = 8 mice per condition; *p = 0.048, **p < 0.001 two-tailed Student’s *t* test), and IgG2b and aVLA4 treated mice (*n* = 6 mice per condition, **p < 0.001 two-tailed Student’s *t* test). Data are expressed as mean ± SEM. **c** Open-field test (10 min) results for IgG2a-, aNK1.1-, IgG2b-, aVLA4-treated mice (*n* = 10 IgG2a and aNK1.1, *n* = 8 IgG2b and aVLA4 mice), and Rag2^−/−^ and Rag2^−/−^γc^−/−^ mice (*n* = 7 per condition) showing total distance traveled (*p = 0.039 IgG2a vs aNK1.1 mice, *p = 0.037 IgG2b vs aVLA4 mice, **p = 0.002 Rag2^−/−^ vs Rag2^−/−^γc^−/−^), percent time spent in the center (**p < 0.001 IgG2a vs aNK1.1 and IgG2b vs aVLA4 mice, **p = 0.022 Rag2^−/−^ vs Rag2^−/−^γc^−/−^), center episodes (*p* = 0.102 IgG2a vs aNK1.1 mice, *p* = 0.151 IgG2b vs aVLA4 mice, *p = 0.036 Rag2^−/−^ vs Rag2^−/−^γc^−/−^), and the total activity (**p < 0.001 IgG2a vs aNK1.1 and IgG2b vs aVLA4 mice; two-tailed Student’s *t* test). Data are expressed as mean ± SEM. **d** Discrimination index analyzed after 1 h (short term memory - STM) and 24 h (long term memory - LTM) in mice treated with IgG2a, aNK1.1, IgG2b and aVLA4 (*n* = 10 IgG2a and aNK1.1, *n* = 8 IgG 2b and aVLA4 mice, **p = 0.003 IgG2a vs aNK1.1, **p = 0.004 IgG2b vs aVLA4, one-way ANOVA), and in Rag2^−/−^ and Rag2^−/−^γc^−/−^ mice (*n* = 7 mice per condition, *p = 0.011 one-way ANOVA). Data are expressed as mean ± SEM. **c**, **d** Open-field test results on distance traveled for IgG2a-treated mice vs Rag2^−/−^ and Rag2^−/−^γc^−/−^ (**p < 0.01 IgG2a vs Rag2^−/−^ mice, **p < 0.001 IgG2a vs Rag2^−/−^γc^−/−^ mice, two-way ANOVA). Object novelty discrimination in IgG2a-treated mice vs Rag2^−/−^ and Rag2^−/−^γc^−/−^ mice (STM **p < 0.001 IgG2a vs Rag2^−/−^ and Rag2^−/−^γc^−/−^ mice; LTM ^**^p < 0.01 IgG2a vs Rag2^−/−^ mice, two-way ANOVA). Data are representative of at least two experiments with similar results. **a**, **d** Figure created with BioRender.com.

To investigate the role of the ILC1/NK cells in modulating brain functions, we treated mice with the NK1.1 antibody (aNK1.1) (Fig. 2a). Since NK cells in C57BL/6 mice largely overlap with ILC1 and NKT for the expression of the NK1.1 and NKp46 cell markers^38^, aNK1.1 treatment affects all these cell populations. Note that in the meninges, the frequency of NKT cells is 1.49 ± 0.12% of CD45^+^ cells (Supplementary Fig. S2a). The efficacy of this treatment in depleting the CD3^−^/NK1.1^+^ cell population in the CNS was already shown^32,34^, and here confirmed for the meningeal compartment (Supplementary Fig. S2b). To assess the impact of aNK1.1 treatment on other immune cells, we determined the frequency and distribution of T cell subsets in the spleen, liver, bone marrow and thymus. Notably, aNK1.1 treatment did not significantly affect T cell differentiation, peripheral frequency and homeostatic properties (Supplementary Fig. S2c) but modified meningeal CD8^+^ T cell frequency (Supplementary Fig. S2d). Profiling aNK1.1-treated mice in a battery of behavioral tests, we report reduction in anxiety-related behavior and impairment in non-spatial memory, suggesting possible cortical and hippocampal deficits^42–44^ (Fig. 2b, d). In detail, in the absence of NK1.1^+^ cells, mice increased both the exploration time in the open arms of the elevated-plus maze test (Fig. 2b), as well as the time spent in the center of the arena in the open-field test (Fig. 2c), thus suggesting reduced anxiety-like response to open spaces and increased explorative behavior. To determine whether the increased exploration during the elevated-plus maze and the open-field tests were associated with abnormal motor activity, we evaluated the total distance traveled in 1 h, and data in Supplementary Fig. S2e revealed no difference between NK1.1^+^ cell-depleted vs control (IgG-treated) mice.

To investigate possible non-spatial memory alterations in aNK1.1-treated mice, we performed the novel object recognition (NOR) test. Results shown in Fig. 2d indicates that, in the absence of NK1.1^+^ cells, mice exhibited a deficit in both the short- (1 h) and long-term (24 h) memory tasks. Note that no sex-based differences were observed in the open-field and NOR tests (Supplementary Fig. S3a, b).

To investigate possible roles played by the infiltrating cell populations in these behavioral changes, we treated mice with antibodies against the integrin very-late antigen (VLA) 4, which is involved in lymphocyte extravasation^22,45^ via binding to the vascular cellular adhesion molecule 1 (VCAM-1) (Fig. 2a). Results obtained indicate that the aVLA4-treatment, which efficiently reduced the number of meningeal NK1.1^+^ cells (Supplementary Fig. S3c) impaired memory and decreased anxiety-like behavior, similarly to aNK1.1-treatment (Fig. 2b, d).

To exclude a possible role of NKT cells in the behavioral modifications observed upon aNK1.1-treatment, *Rag2*^−/−^ (free from NKT, T and B cells) and *Rag2*^−/−^*γc*^−/−^ mice (free from NKT, T, B cells and ILCs) were tested in these same experimental tasks. Note that the *Rag2*^−/−^ mice have different behavior in the open-field and in the NOR tests, confirming previous evidence of the role of adaptive immune cells in mice behavior^17–19^. When compared with *Rag2*^−/−^ mice, the *Rag2*^−/−^*γc*^−/−^ mice performed similarly to aNK1.1-treated mice (vs IgG-treated wt mice) both in the NOR and in the open-field tests, providing further evidence for a role of ILC1/NK cells in behavioral modulation (Fig. 2c, d).

Overall, these results demonstrate that the removal of innate immune CD3^−^/NK1.1^+^ cells modifies mouse behavior, reducing anxiety-like behavior and non-spatial memory abilities.

### NK cells/ILC1 are involved in the regulation of cortical GABAergic activity through interferon-γ, affecting memory

**Fig. 3:**
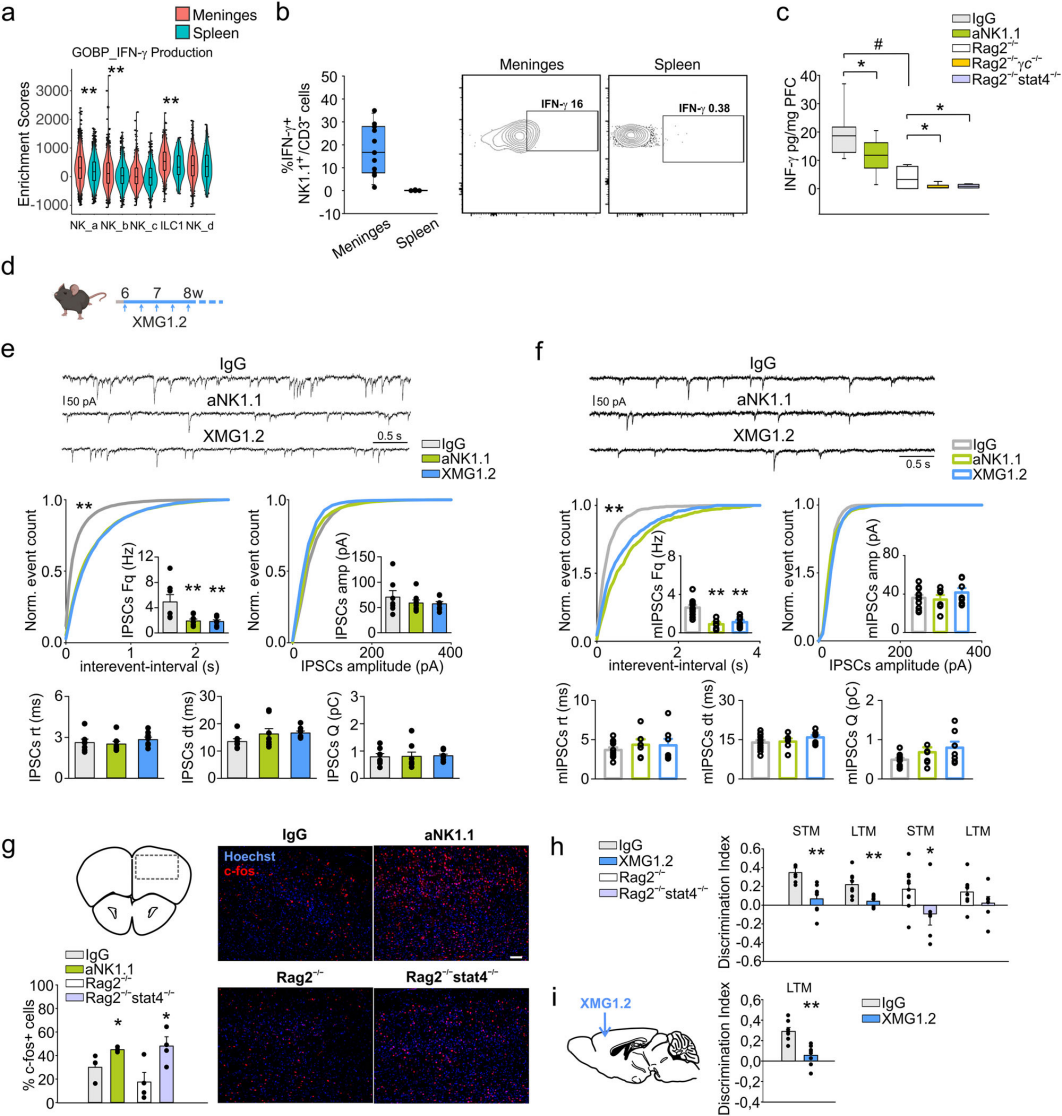
ILC1-NKcells derived-IFN-γ regulates non spatial memory. **a** Violin plot of the distribution of genes related to IFN-γ production in different clusters and tissue (*n* = 50 mice **p < 0.01 Kruskal–Wallis post-hoc Dunn test). **b** FACS analysis of frequency of IFN-γ^+^ cells in the CD3^−^/NK1.1^+^ cell population obtained from the meninges and spleen (*n* = 11). Right: representative plot of meningeal and splenic frequency of CD3^−^/NK1.1^+^ cells. **c** Level of IFN-γ protein in the PFC of mice treated with IgG or aNK1.1 (*n* = 9 mice per condition, *p = 0.033 two-tailed Student’s *t* test), and Rag2^−/−^, Rag2^−/−γ^c^−/−^ and Rag2^−/−^stat4^−/−^ mice (*n* = 7 mice per group, *p = 0.013 two-tailed Student’s *t* test; IgG-treated mice vs Rag2^−/−^^#^*p* < 0.001 two-tailed Student’s *t* test). For boxplots (**a–c**) the center line, boxes and whiskers represent the median, inner quartiles, and rest of the data distribution, respectively. **d** Scheme of XMG1.2 treatment in C57BL/6 mice. **e** Top: Representativ**e** traces of sIPSCs recorded in PFC L5 pyramidal neurons obtained from mice chronically treated with IgG, aNK1.1, or XMG1.2. Bottom: Cumulative distribution and bar graph (inset) of interevent-interval, amplitude values, rise time (rt), decay time (dt) and mean charge (Q) mean values of sIPSCs obtained from 7, 8, and 8 cells recorded from mice treated with IgG, aNK1.1, and XMG1.2, respectively (one-way ANOVA **p < 0.001, Ks = 0.95). The inset bar graph represents the mean frequency values recorded in the same cells (one-way ANOVA **p = 0.005). Data are expressed as mean ± SEM. **f** Top: Representative traces of mIPSCs recorded in L5 pyramidal neurons obtained from mice chronically treated with IgG, aNK1.1, or XMG1.2. Bottom: Cumulative distribution of interevent-interval, amplitude values, rise time (rt), decay time (dt) and mean charge (Q) mean values of mIPSCs obtained from 11, 7, and 6 cells recorded from mice treated with IgG, aNK1.1, and XMG1.2, respectively (**p < 0.001, Ks = 0.68). The inset bar graph represents the mean frequency values recorded in the same cells (*p < 0.001). Data are expressed as mean ± SEM. **g** Analysis of c-fos^+^ cells in the PFC of mice treated with IgG or aNK1.1 and Rag2^−/−^ or Rag2^−/−^stat4^−/−^ mice expressed as % of total nuclei (*n* = 4 mice per group, *p < 0.034 two-tailed Student’s *t* test). Representative immunofluorescences on the right. Error bars show mean ± SEM. Scale bar: 100 μm. **h** Discrimination index in NOR test analyzed after 1 h (STM) and 24 h (LTM) in mice treated with IgG and XMG1.2 (*n* = 8 mice per condition; **p < 0.001 one-way ANOVA), and Rag2^−/−^ (*n* = 11) and Rag2^−/−^stat4^−/−^ (*n* = 7) mice (*p = 0.034 one-way ANOVA). Data are expressed as mean ± SEM. **i** Left: Scheme of XMG1.2 intracerebral administration in C57BL/6 mice. Right: Discrimination index in NOR test analyzed in mice with intra-hippocampal administration of IgG or XMG1.2 (*n* = 7 mice per condition; **p < 0.004 one-way ANOVA). Data are expressed as mean ± SEM. (**d**, **g**, **i**) Figure created with BioRender.com.

To address the molecular mechanisms underlying the behavioral effects modulated by NK cells and ILC1, we investigated the role of IFN-γ. This cytokine was recently associated with the modulation of inhibitory neuronal activity^22^, while GABAergic circuits in the prefrontal cortex (PFC) affect memory formation^46^. Single cell RNA-seq analysis revealed that meningeal NK cells and ILC1 upregulated the expression of genes related to IFN-γ pathways (production and signaling) (Fig. 3a; Supplementary Fig. S4a), while only meningeal NK_b, NK_d and ILC1 clusters expressed *Ifn*-*g* (Fig. 1g). FACS analysis of meningeal T lymphocytes and CD3^−^/NK1.1^+^ cells demonstrate the spontaneous IFN-γ expression by these cells (Fig. 3b, Supplementary Fig. S4b). In contrast, splenic CD3^−^/NK1.1+ cells do not express IFN-γ (Fig. 3b), suggesting a tissue-specific regulation.

Accordingly, STAT4, which is involved in *Inf-g* gene transcription in ILC1 and NK cells^25^ is more expressed by meningeal than splenic NKp46^+^ cells (Supplementary Fig. S1e). Low levels of STAT4 expression were also found in myeloid cells (Supplementary Fig. S4c).

In the PFC region, the basal IFN-γ protein level was reduced in aNK1.1- compared to IgG-treated mice and in *Rag2*^−/−^*γc*^−/−^ compared to *Rag2*^−/−^ mice (Fig. 3c). Similarly, low levels of IFN-γ were observed in *Rag2*^−/−^S*tat4*^−/−^ mice, where IFN-γ transcription is specifically impaired in ILC1 and NK cells^25^ (Fig. 3c), further confirming the specific involvement of these cells in modulating CNS IFN-γ levels. (Fig. 3c). These findings provide evidence for the contribution of ILC1/NK cells in sustaining the homeostatic levels of IFN-γ in the PFC and in the meninges. To investigate possible effects of basal IFN-γ on the neurotransmission in the PFC, we recorded the inhibitory currents in the pyramidal neurons of the PFC layer 5, in IgG-, aNK1.1- or XMG1.2- (an anti IFN-γ antibody) i.p. treated mice (Fig. 3d). Patch clamp recording revealed that both XMG1.2 and aNK1.1 treatment reduced the frequency of spontaneous inhibitory post-synaptic currents (sIPSCs), with no changes in current amplitude or other sIPSC kinetics parameters (Fig. 3e). To investigate if this effect was due to a modulation of GABA release or to a diminished local network activity, we recorded miniature inhibitory postsynaptic currents (mIPSCs) from the same cells. Both aNK1.1 and XMG1.2 reduced the event frequency, without affecting other parameters (Fig. 3f), indicating that ILC1/NK cells-derived IFN-γ can contribute to the homeostasis of presynaptic GABA release in the PFC. Consistently, aNK1.1-treated mice (compared to wt-IgG) and *Rag2*^−/−^*Stat4*^−/−^ mice (compared to *Rag2*^−/−^), displayed increased c-fos^+^ (active) neurons in the PFC, after the NOR test (Fig. 3g). and both XMG1.2-treated, as well as *Rag2*^−/−^*Stat4*^−/−^ mice had lower indexes in the NOR test (Fig. 3h).

To investigate possible indirect peripheral effects induced by IFN-γ blockade, we injected XMG1.2 intracranially: this local treatment reproduced the effects of peripheral administration on the NOR assay (Fig. 3i), strongly indicating a direct effect of brain IFN-γ in the regulation of non-spatial memory. Together, these data confirm the role of IFN-γ as molecular link between ILC1/NK cells and memory. Interestingly and in accordance with previous evidence^22^, IFN-γ depletion did not affect mice behavior in the open-field test (Supplementary Fig. S4d), suggesting different molecular pathways for the anxiety-like behavior regulated by NK cells and ILC1.

### Acetylcholine released by NK cells/ILC1 triggers the activation of neuronal circuits and anxiety-like behavior

**Fig. 4:**
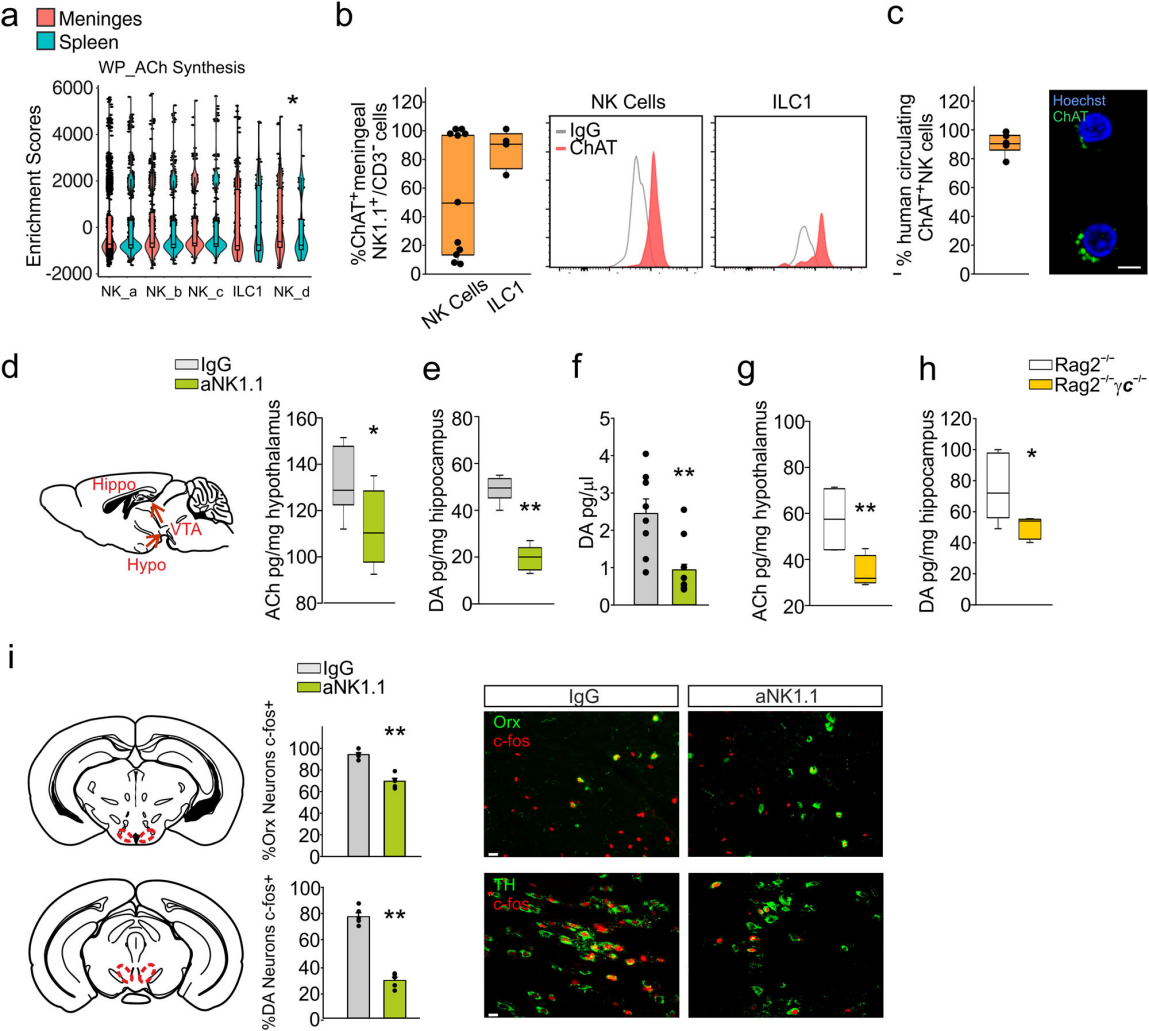
ILC1-NKcells derived-Ach regulates anxiety like behavior. **a** Violin plot of the distribution of genes related to ACh synthesis in different clusters and tissue (*n* = 50 mice *p < 0.05 Kruskal–Wallis post-hoc Dunn test). **b** FACS analysis of the frequency of ChAT^+^ cells in the CD3^−^/NK1.1^+^/CD49b^+^ (NK) cell (*n* = 6) and CD3^−^/NK1.1^+^/CD49a^+^ cell (ILC1) (*n* = 4) populations obtained from the meninges. **c)** Immunofluorescence analysis of the percentage of ChAT^+^ NK cells collected from the blood of healthy donors (*n* = 6). Scale bar: 5 μm. For boxplots (**a**–**c**) the center line, boxes and whiskers represent the median, inner quartiles, and rest of the data distribution, respectively. **d** Left: Scheme of ACh intracerebral administration in C57BL/6 mice. Right: Expression of ACh in the hypothalamus of mice treated with IgG or aNK1.1 (*n* = 6 mice per condition, *p = 0.036 two-tailed Student’s *t* test). Left: Scheme of hypothalamus-VTA-hippocampus circuit. **e** Expression of DA in the hippocampus of mice treated with IgG or aNK1.1 (*n* = 6 mice per condition, **p < 0.001 two-tailed Student’s *t* test). **f** Microdialysis of basal DA in the hippocampus of IgG- (*n* = 6) or aNK1.1-treated (*n* = 9 mice, **p = 0.005 one-way ANOVA). **g** Expression of ACh in the hypothalamus of Rag2^−/−^ and Rag2^−/−^γc^−/−^ mice (*n* = 6 mice per group, **p = 0.002 two-tailed Student’s *t* test). **h** Expression of DA in the hippocampus of Rag2^−/−^ and Rag2^−/−^γc^−/−^ mice (*n* = 6 mice per condition, *p = 0.019 two-tailed Student’s *t* test). For boxplots (**d**–**h**), the center line, boxes and whiskers represent the median, inner quartiles, and rest of the data distribution, respectively. **i** Immunohistochemistry demonstrating c-Fos expression in orexin hypotalamic neurons and in VTA dopaminergic neurons following behavioral test in IgG- or aNK1.1-treated mice. Center: quantification of c-Fos^+^ cells express as percentage of total Orx^+^ or TH^+^ neurons in the different areas (*n* = 6 mice per condition; **p < 0.001, two-tailed Student’s *t* test. Scale bar: 50 μm). Data are expressed as mean ± SEM. (**d**, **i**) Figure created with BioRender.com.

It was previously reported that, in pathological conditions, NK cells interact with neurons and microglia through the release of ACh^47^. To understand the mechanisms underlying the modulatory effect of NK1.1^+^ cells on anxiety-like behavior in mice, we investigated whether the ACh released by ILC1/NK cells could be involved. scRNA-seq analysis reveals that meningeal CD3^−^/NK1.1^+^ cells expressed higher levels of a number of genes related to ACh synthesis and signaling pathways, compared to splenic CD3^−^/NK1.1^+^ cells (Fig. 4a; Supplementary Fig. S5a). Murine meningeal CD3^+^ T cells, NKT cells, ILC1 and NK cells, and human blood-derived NKp46^+^ cells express choline acetyltransferase (ChAT) (both protein and mRNA) (Fig. 4b, c; Supplementary Figs. S5b, c and S9). When cultured in vitro, these cells release ACh and respond to PMA/ionomycin and IL-15 stimulation, or to microglial co-culture with increased ACh release (Supplementary Fig. S5d).

**Fig. 5:**
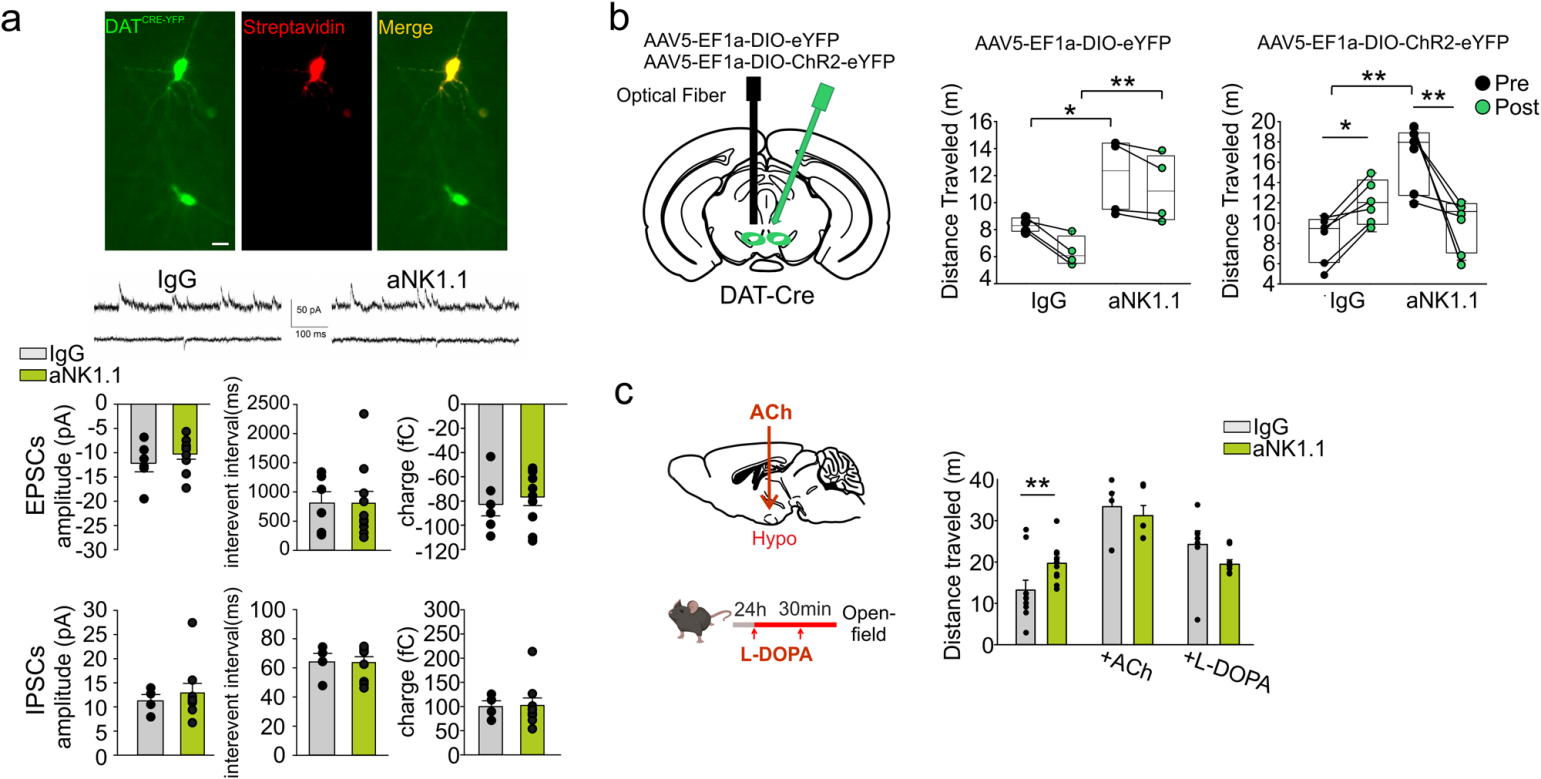
Optogenetically reactivated VTA restores aNK1.1-mice behavior. **a** Middle: Representative traces of sIPSCs (upper) and sEPSCs (lower) recorded in VTA DAT^CRE-yfp^ neurons (top) obtained from mice treated with IgG and aNK1.1. Scale bar: 20 μm. Bottom: Bar graph of interevent-interval, amplitude values, and charge mean values of sIPSCs and sEPSCs obtained from 6 cells recorded from mice. **b** Left: scheme of virus injection and optic fiber implantation. Right: in the open-field test, ChR2:YFP-expressing IgG-treated mice increase the distance traveled, in contrast the aNK1.1-treated mice displayed a reduction in the explorative behavior (after 5 min in the new arena light stimulation is carried out, maintained for additional 5 min) (*n* = 6 mice per treatment, one-way ANOVA *p < 0.05 **p < 0.01). **c** Left: Scheme of ACh and L-DOPA administration in C57BL/6 mice. Right: Open-field test results for IgG- and aNK1.1-treated mice after the intra-hypothalamic administration of ACh or i.p. injection with L-DOPA as indicated (left), showing total distance traveled (*n* = 10 IgG- and aNK1.1 mice; *n* = 6 ACh-IgG- and ACh-aNK1.1 mice; **p < 0.001 two-way ANOVA; L-DOPA IgG vs aNK1.1 **p = 0.002, two-way ANOVA). Data are expressed as mean ± SEM. **b**, **c** Figure created with BioRender.com.

We then investigated the activation of the lateral hypothalamus (LH)-ventral tegmental area (VTA)- hippocampus circuit upon the open-field test. This neuronal network has been associated with several behavioral tasks: the orexin neurons in the LH respond to ACh^48^ and, in turn, stimulate dopamine (DA)-release in the hippocampus by VTA dopaminergic neurons, regulating the explorative and the anxiety-like behavior^49^. We describe that aNK1.1-treated mice had reduced level of ACh in the hypothalamus (Fig. 4d), while no differences in the serum level of ACh was observed among wt-IgG and aNK1.1- or aVLA4-treated mice, suggesting a local ACh release (Supplementary Fig. S5e). Furthermore, aNK1.1-treated mice had reduced level of dopamine (DA) in the hippocampus (Fig. 4e, f). The involvement of NK1.1^+^ cells in the activation of LH-VTA-hippocampus neuronal circuit was further confirmed by the reduction of hypothalamic ACh and hippocampal DA levels in *Rag2*^−/−^*γc*^−/−^ *vs Rag2*^−/−^ mice (Fig.4g, h), and by a reduced c-fos staining of hypothalamic orexin and VTA dopaminergic neurons in the aNK1.1-treated mice, upon open-field testing (Fig. 4i). Note that, in comparison with *wt*, *Rag2*^−/−^ mice showed different basal levels of neurotransmitters (Fig. 4d–h), as well as increased traveled distance in the open-field test and impaired memory in the NOR test (Fig. 2c, d), possibly due to additional modulation by other immune cells. We can exclude that the observed effects could be due to direct alteration of the synaptic drive onto dopaminergic neurons, since no differences were observed in the sIPSCs and spontaneous excitatory post-synaptic currents (sEPSCs) recorded on VTA dopaminergic neurons in aNK1.1- and IgG-treated mice (Fig. 5a). To better elucidate the role of VTA neurons in this modulatory mechanism, we took advantage of AAV-mediated ChR2:YFP expression to control dopaminergic DAT^CRE-yfp^ neuron activation (Supplementary Fig. S6a). Data in Fig. 5b demonstrated that the direct optogenetic activation of these neurons restored the explorative behavior of aNK1.1-treated mice in the open-field test, increasing the level of DA in hippocampus (Supplementary Fig. S6b). DA levels in the hippocampus of aNK1.1-treated mice were also re-established either by ACh in the hypothalamus (cannula infusion 30 min before the open-field test), or by L-DOPA (two i.p. injections, 1 day and 30 min before the test) (Supplementary Fig. S6c, d). Both treatments restored the distance traveled and the time spent in the center of the arena to values observed in IgG-treated mice (Fig. 5c), with no effect on the NOR test (Supplementary Fig. S6e, f), confirming the involvement of different ILC1/NK cells-mediated mechanisms for memory impairment.

## Discussion

The mechanisms of bidirectional communication and reciprocal modulation among the CNS and the immune system are still far to be completely understood^1–5,12–15,20–22,50^. Here we demonstrate that NK cells and ILC1 actively regulate neuronal function, modulating the cortical GABAergic and the hypothalamic orexinergic neurotransmission. We describe that ILC1/NK cell-derived IFN-γ and ACh participate in the regulation of specific brain circuits involved in anxiety-like and memory in mice, discovering a previously unknown neuromodulatory role for innate immune cells under physiological conditions.

Previous reports described the presence of NK cells in the CNS under healthy conditions^24,25^. However, the number and phenotype of NK cells in the CNS change in different pathological conditions. In MS patients and in mice upon EAE induction a different distribution of NK cell subsets has been reported^26,28,51^, while ILC1 acquire a protective phenotype^24^. The frequency of NK cells in the CNS also changes in brain tumors, where the immunosuppressive microenvironment reduces NK cell ability to recognize and kill tumoral cells^30–32,52,53^. In mouse models of ALS, reducing NK cells infiltration in the CNS slows disease progression^34^. Signaling through specific chemokine receptors could be involved in ILC1 and NK cells entry into the CNS upon damages^26,28,36^, but further studies will be necessary to understand the potential role of brain-produced chemotactic signaling through CXCR6 (ILC1) and CXCR4 (ILC1 and NK cells), among others.

Thus, a better knowledge of ILC1 and NK cells present in the meninges under healthy conditions could be fundamental to define their role in the interaction with the brain. Our scRNAseq analyses reveal a reduced expression of *Itga4* and an increase of *Irf8* and *Cxcr6* in meningeal ILC1, while *vegfa, Ifn-g, Cxcr4* and *Smad7* were also higher in NK cells. Using the SCENIC algorithm, we describe higher *RORa* activity in meningeal ILC1, and higher *Eomes* and *Klf2* activity in splenic NK cells. These TFs are involved in ILC1 and NK cells maturation^54,55^. On the other hand, meningeal NK cells showed higher activity for the TFs of the Fos and Jun families as shown for tissue-resident NK cells^56,57^. These differences could be due to different tissue origins^39^, to the innate lymphocyte plasticity^40^ or could highlight specific meningeal cell functions in comparison with peripheral cells^41^, worth to further investigation.

In this paper we describe that the removal of the ILC1/NK cells modifies mice behavior, reducing anxiety-like behavior and non-spatial memory abilities. No evidence of an involvement of NK cells or ILC1 on emotional behaviors have been reported in humans so far, while alterations of NK cell number and function have been described in altered psychiatric conditions, such as in major depression and stress^58,59^. Possible alterations in NK cell number or their activation states (or maturation), with effects on behavior, synaptic GABAergic activity and, in general, inhibition and excitation balance could be due to an alteration of gut microbiota^60^, infections, immune challenges and physical exercise^61,62^.

Meningeal adaptive immunity-derived IFN-γ modulates GABAergic neurotransmission as well as learning and social behaviors^22^. We report that in the meninges, the ILC1/NK cells showed a higher level of IFN-γ and STAT4 in comparison to splenic ILC1/NK cells. Although we detect very low level of STAT4 expression in meningeal myeloid cells, we cannot exclude an induction of STAT4 upon myeloid cell activation^63^. We report that NK1.1^+^ cells depletion reduced the sIPSC frequency of cortical pyramidal neurons potentially in an IFN-γ-dependent way, being similarly reduced by aNK1.1 or XGM1.2 treatment, with no effect on current amplitude, suggesting a presynaptic modulatory effect on GABAergic cortical neurons. The effects of IFN-γ on GABAergic neurotransmission and on plasticity were described in hippocampal and prefrontal cortical neurons^22,23,64^, but we cannot exclude that the effects on GABAergic neurotransmission are mediated by glial cells or perivascular macrophages^65,66^. It has been demonstrated that local cytokines in the meningeal niche, such as IL-1 and IL-12, could induce the production of IFN-γ by NK cells^67,68^, but further studies will be necessary to better understand the molecular mechanisms that modulate IFN-γ production in the meninges.

IFN-γ depletion also specifically reduced mice performance in the NOR test, similar to aNK1.1 treated mice, supporting the involvement of the NK1.1^+^ cells-IFN-γ signaling specifically in memory behavior, although IFN-γ produced by different subgroups of meningeal immune cells, or from different brain compartments (i.e. choroid plexus or parenchyma) and/or the periphery could play a role.

IFN-γ depletion, in turn, did not affect the anxiety-like behavior regulated by ILC1/NK cells, suggesting specific effects on memory-related neuronal networks^22,64,65^.

Previous reports described that activated NK cells release ACh^47^. Here, we demonstrated that meningeal NK cells release ACh in physiological condition. Subpopulation of T cells in the spleen and other tissues express ChAT, and release ACh in response to vagus nerve activation, modulating innate immune response and blood pressure^69,70^. By scRNA-seq, we observed that meningeal NK cells and ILC1 upregulated genes related to ACh synthesis and that murine and human NK cells express ChAT. Mice depleted of NK1.1^+^ cells had reduced ACh levels in the hypothalamus, with no variation in serum ACh, and this reduction affected DA levels in the hippocampal region through the reduction of dopaminergic VTA neurons activity. The involvement of the dopaminergic VTA neurons in the explorative and anxiety-like behavior of mice is well known^42–44^, and here we link for the first time the activation of this neuronal network to ILC1/NK cells under basal, steady state conditions.

Moreover, despite the reduction of meningeal NK cells/ILC1 upon the administration of VLA4 antibody reproduces the effects of NK1.1^+^ cell depletion on mice behavior, we cannot exclude a possible role also played by the peripheral innate immune NK1.1^+^ cells, or the NK cells/ILC1 resident in the brain (such as in the choroid plexus^24,25^). In conclusion, here we describe specific immune- neuronal networks and identify IFN-γ and ACh as two soluble mediators involved in the basal modulation of exploratory, anxiety-like and learning behavior in mice.

## Methods

### Materials

All cell culture media, fetal bovine serum (FBS), goat serum, penicillin G, streptomycin, secondary Abs, and Hoechst (catalog #33342, RRID:AB_10626776) were from GIBCO Invitrogen (Carlsbad, CA, USA). Phosphate buffered-saline (PBS) tablet (#P4417), Bovine Serum Albumine (BSA) and deoxyribonuclease I, were from Sigma-Aldrich (Milan, Italy). NKp46 (M20) (#sc-18161, RRID:AB_2149152) antibody (Ab) was from Santa Cruz biotechnology (Santa Cruz, CA). RNeasy Mini Kit was from Qiagen (Hilden, Germany). Fluorochrome-conjugated anti-CD45 (clone 104), -CD3 (clone 145-2C11), -CD4 (clone RM4-5), -CD8 (clone 53-6.7), -CD44 (clone IM7), -CD62L (clone MEL-14), -NK1.1 (clone pk136), -NKp46 (clone 29A1.4), -CD19 (clone 6D5), -CD49a (clone Ha31/8), -CD49b (clone DX5), -CD11b (clone M1/70), Gr1 (RB6-8C5), Ly6C (clone HK1.4), and F4/80 (BM8) mAbs were from eBioscience Inc., Biolegend or BD biosciences (San Diego, CA).Rat anti–IFN-γ monoclonal antibody (clone: XMG1.2) (Cat# BE0055, RRID:AB 1107694), anti-NK1.1 (Cat# BE0036, RRID:AB_1107737), and anti-VLA4 (CD49d/Natalizumab) (#BE0071, RRID:AB_1107657) were from Bioxcell (West Lebanon, USA).

### Animals

Experiments described in the present work were approved by the Animal Welfare Body of Sapienza University and the Italian Ministry of Health (authorization n° 775/2020-PR; n° 70/2022; n° 356/2023-PR) in accordance with the guidelines on the ethical use of animals from the European Community Council Directive of September 22, 2010 (2010/63/EU), and from the Italian D.Leg 26/2014. All possible efforts were made to minimize animal suffering, and to reduce the number of animals used per condition by calculating the necessary sample size before performing the experiments. All studies were performed using adult male and female mice at the indicated ages. C57BL/6 (IgG *wild type-wt*), Rag2^−/−^ (B6.Cg-*Rag2*^*tm1.1Cgn*^/J, RRID:IMSR_JAX:008449), Rag2^−/−^*γc*^−/−^ (C;129S4-*Rag2tm1.1Flv Il2rgtm1.1Flv*/J, RRID:IMSR_JAX:014593), and DAT^CRE-yfp^ (B6.SJL-*Slc6a3*^*tm1.1(cre)Bkmn*^/J, RRID:IMSR_JAX:006660)mice were obtained from Jackson Laboratory (Bar Harbor, ME, USA) and from Charles River (Calco, Italy). Rag2^−/−^stat4^−/−^ mice were housed in the Sapienza University animal facility. Mice were housed in standard breeding cages at constant temperature (22 ± 1 °C) and relative humidity (50%), with a 12:12 h light:dark cycle (light on 07.00–19.00 h). Food and water were available ad libitum. Microbiological analyses were routinely (each 3–4 months) performed and defined endemic Norovirus in our conventional animal facility.

### Human samples

Healthy donors stratified for age and sex were enrolled, recruited from the Rare Neuromuscular Diseases Centre of Umberto I Hospital in Rome (ref 3314/25.09.14, protocol n. 1186/14). Peripheral blood mononuclear cells (PBMCs), freshly isolated from healthy donors by lymphoprep (Nycomed AS, Oslo, Norway) were frozen and stored at −80° for up to 2 months. The day before the experiment, cells were thawed and kept in culture over night for recovering. Then, dead cells were removed, and cells were stained with anti-NKp46 and -ChAT antibodies for flow cytometry analysis or were purified using NK cell isolation kit (Miltenyi Biotec). Purified cells were layered on poly-lysine-coated slides for ChAT staining and fluorescence microscopy analysis.

### Meningeal dissection

Mice, anesthetized with Zoletil and Rompun, were transcardially perfused with ice-cold PBS to remove circulating immune cells. To isolate the meninges, the skull cap was removed by performing two lateral incisions starting from the foramen magnum until the nasal bone. The brain was removed taking care to remove any visible remnants, and meninges (mainly the dura layer^6^) were detached from the skull. Meninges were collected in Eppendorf vials containing 0.5 ml of DMEM.

### Isolation of CD3^−^/NK1.1+ cells

The meninges and spleens of C57BL/6 mice were enzymatically digested in DMEM collagenase 1 mg/ml for 30 min at 37 °C. Digestion reaction was stopped by addition of DMEM 10% FBS and dissociation into single cell suspension is finalized by mechanical disruption on 70 μm cell strainer (Falcon, Beckton Dickinson). The cell suspensions were washed and resuspended in staining buffer (PBS without Ca^2+^ Mg^2+^, BSA 0,5%, EDTA 2 mM and NaN_3_ 0,025%), tagged with BD single cell multiplexing kit, mixed and stained with Zombie Violet for 15 min at room temperature for exclusion of dead cells and anti-CD16/32 blocking mAb (clone 24G2) for 10 min at 4 °C followed by incubation with anti-CD45, -CD3, -NK1.1 mAbs for 25 min. CD45^+^CD3^−^NK1.1^+^ gated cells were sorted into PBS buffer 2% BSA 0,5 mM EDTA using a FACSAriaII (BD Biosciences) equipped with a 488, 561 and a 633 nm laser and FACSDiva software (BD Biosciences version 6.1.3), and retained on ice. To reduce stress, cells were isolated in gentle FACS-sorting conditions using a ceramic nozzle of size 100 μm, a low sheath pressure of 19.84 pound-force per square inch (psi) that maintain the sample pressure at 18.96 psi and an acquisition rate of maximum 1500 events/s. FACS-sorted cells were confirmed to be 98% pure prior to RNA extraction. To evaluate intracellular markers, cells were fixed and permeabilized by using BD Cytofix/Cytoperm^**TM**^ Fixation/Permeablization Kit. All cells were analyzed by flow cytometry using a FACSCanto II (BD Biosciences), and data were elaborated using FlowJo software (Becton Dickinson). The gating strategies were indicated in Supplementary Figs. 7–9.

### Flow cytometry

Single cell suspension was isolated from spleen and meninges as described in “Isolation of NK1.1^+^CD3^−^ cells” section. Cells were stained with fixable viability dye 780 to exclude dead cells and with anti-CD45 along with fluorochrome conjugated mAb specific for immune cell populations that were identified as NK1.1^+^CD3^−^ (for NK cells), CD3^+^CD8^+^ (for CD8^+^ T cells), CD3^+^CD4^+^ (for CD4^+^ T cells), CD19^+^ (for B cells), CD11b^+^Gr1^high^ (for neutrophils), CD11b^+^Gr1^+^Ly6C^high^ (monocytes) or CD11b^+^GR1^-^F4/80^+^ (macrophages). Among T cells, naïve cells were gated as CD44^−^CD62^+^ and effector/memory as CD44^+^CD62^−^. To evaluate IFN-γ expression, cell suspension was fixed and permeabilized by using BD Cytofix/Cytoperm™ Fixation/Permeablization Kit and stained with isotype or anti-IFN-γ mAb. For T cells, cells were stimulated with PMA/ionomycin for 2 h before staining. To evaluate STAT4 and Chat expression, cells were fixed and permeabilized using FoxP3/ Transcription Factor Staining Buffer Set, stained with isotype control, anti-STAT4 or anti-CHAT polyclonal primary antibodies followed by PE Donkey anti-rabbit IgG (Poly4064) labeling. All cells were analyzed by flow cytometry using a FACSCanto II (BD Biosciences), and data were elaborated using FlowJo software (Becton Dickinson).

### Single-cell transcriptomics

Cells from meninges and spleens were sequentially labeled using Single Cell Labeling with the BD Single-Cell Multiplexing Kit (BD Biosciences, # 633793). Briefly, cells were labeled with sample tags and each sample was washed twice with FACS buffer. Samples were counted in a hemocytometer (InCyto, DCH-NO1-5) staining the cells with Calcein AM (Thermo Fischer Scientific, #C1430) and Draq7^TM^ (BD Biosciences, # 564904). Samples were pooled and resuspended in cold BD Sample Buffer (BD Biosciences) to achieve approximately 30.000 cells in 620 μL. Single cells from the pooled sample were isolated using Single Cell Capture and cDNA Synthesis with the BD Rhapsody Express Single-Cell Analysis System following the manufacturers protocol (BD Biosciences). After priming the nanowell cartridges, the pooled sample was loaded onto BD Rhapsody cartridge and incubated at room temperature. Cell Capture Beads (BD Biosciences) were prepared and then loaded onto the cartridge. According to the manufacturers protocol, cartridge was washed, cells were lysed, and Cell Capture Beads were retrieved and washed prior to performing reverse transcription and treatment with Exonuclease I. cDNA Libraries were prepared using mRNA whole transcriptome analysis (WTA) and Sample Tag library preparation protocol (BD Biosciences).

The protocol allows to screen RNA expression of single cell using a 3′ WTA approach for samples that have been labeled with the BD Single-Cell Multiplexing Kit.

PCR products were purified, and WTA mRNA PCR products were separated from sample tag products with double-sided size selection using SPRIselect magnetic beads (Beckman Coulter, #B23318). Quality and quantity of PCR products were determined by using an Agilent 2200 TapeStation with High Sensitivity D5000 ScreenTape (Agilent). WTA mRNA and sample tag products were diluted to 1 ng/mL to prepare final libraries. Final libraries were indexed using PCR (9 or 6 cycles). Index PCR products were purified using SPRIselect magnetic beads. Quality of final libraries was assessed by using Agilent 2200 TapeStation with High Sensitivity D5000 ScreenTape and quantified using a Qubit Fluorometer using the Qubit dsDNA HS Kit (ThermoFisher, #Q32854). Final libraries were diluted to 4 nM and multiplexed for paired-end (2x75bp) sequencing on a NovaSe +q6000^TM^ sequencer (Illumina). Sequence reads were aligned to the reference mouse genome mm10 (UCSC), following generation of barcode-gene matrices via the pipeline “BD Rhapsody™ WTA Analysis” from SevenBridges (https://www.sevenbridges.com/). The R package Seurat v4.05 was used under RStudio v4.1.5 for data trimming, unsupervised clustering and visualization according to the authors guidelines^71^. In details, in order to remove poor quality cells, doublets and stressed cells, we removed cells with a gene number less than 200, or higher than the 93rd quantile. Mitochondrial gene ratio was calculated to filter out low-quality cells (mitochondrial ratio ≥25%). Each experimental replicate was merged with the Seurat “integration” function^72^ and the resulting dataset was further processed with “SCTransform” for normalization and data scaling^73^. Highly variable genes (HVG, *n* = 3000) were also identified with the “SCTransform” function. The HVGs were used as input for principal component analysis (PCA). The first 30 PCAs were utilized in the subsequent analysis. With them, cells were then embedded by Umap plot with a resolution of 0.5. To assign cell identities, we applied the “FindAllMarkers” to identify differentially expressed genes (DEGs) among all genes by using Wilcoxon rank sum test. We selected only genes that showed 1) a minimal expression (min.pct ≥25%) in at least one cluster, 2) an adjusted *p*-value ≤ 0.05, 3) an average log2-fold change (logFC) ≥ 0.25. For the definition of cell commitment, dedicated immune signature module scores were added to each cell by the “AddModuleScore” function according to specific lists of known cell type markers deriving from Robinette et al., 2015^74^. The ssGSEA analysis on our scRNA dataset was performed with the “escape” package (v1.6.0) utilizing the murine gene sets “H”, “C2”, “C5”, “C7” from the Molecular Signature Database (v7.4). The SCENIC transcription factor inference was performed using the package SCENIC (v1.2.4) following the recommended guidelines^38^.

### Isolation of neurons (NeuN-positive cells) and extraction of total RNA

The isolated motor cortex was cut in small pieces and single-cell suspension was achieved by enzymatic digestion in trypsin (0.25 mg/ml) solution in HBSS. The tissue was further mechanically dissociated using a wide-tipped glass pipette and the suspension applied to a 70 μm nylon cell strainer. Cells, obtained after a three-step Percoll gradient^32,34^ were stained with anti-NeuN Ab (1:1000) at 4 °C for 30 min and isolated using a FACSAria II (BD Biosciences). Cell purity was at least 94%, as verified by flow cytometry and PCR analysis^32,34^. After cell sorting, total RNA was isolated by RNeasy Mini Kit and processed for real-time PCR.

### Real-time PCR

Reverse transcription reaction was performed in a thermocycler (MJ Mini Personal Thermal Cycler; Biorad) using IScript TM Reverse Transcription Supermix (Biorad) according to the manufacturer’s protocol, under the following conditions: incubation at 25 °C for 5 min, reverse transcription at 42 °C for 30 min, inactivation at 85 °C for 5 min. Real-time PCR (RT-PCR) was carried out in a I-Cycler IQ Multicolor RT-PCR Detection System (Biorad) using SsoFast EvaGreen Supermix (Biorad). The PCR protocol consisted of 40 cycles of denaturation at 95 °C for 30 s and annealing/extension at 60 °C for 30 s. For quantification analysis, the comparative Threshold Cycle (Ct) method was used. The Ct values from each gene were normalized to the Ct value of *Gapdh* in the same RNA samples. Relative quantification was performed using the 2^ΔDDCt^ method and expressed as fold change in arbitrary values. The primers used were *gapdh* fw 3′-TCGTCCCGTAGACAAAATGG-5′, rev 3′-CATGCCAGTGAGCTTCCCGTT-5′; chat fw 3′-CCATTGTGAAGCGGTTTGGG-5′, rev 3′-GCCAGGCGGTTGTTTAGATACA-5′.

### Mice treatment

Starting at 6 weeks of age, C57BL/6J and DAT^CRE-yfp^ mice were randomly grouped for the treatments. NK cell depletion was performed using a blocking Ab against NK1.1, which recognizes an epitope of the NKR1Pc-activating receptor (PK136). Mice were i.p. injected with 200 μg (in 100 μl) of anti-NK1.1 Ab every 2 days the first week, every 4 days the second week and then repeated once a week until the age described in the text, at least three weeks. NK cell depletion from the blood sample and meninges was monitored by FACS. For Ab anti-IFNγ treatment, mice were treated with 200 μg of rat XMG1.2, by i.p. injection repeated every 5 days until the mice were sacrificed. For Ab anti-VLA4 administration, mice were i.p. treated with 200 μg, every 4 days for three weeks. For each experiment, control mice were treated with the corresponding control IgG, with no differences between untreated wt and IgG-treated wt mice.

To increase the DA levels specifically in the brain, mice were injected intraperitoneally (i.p.) with two 10 mg/kg doses of L-DOPA (D1507, Sigma-Aldrich), in combination with 12.5 mg/kg benserazide (B7283, Sigma-Aldrich; a peripheral dopa decarboxylase inhibitor) 1 day and 30 min before the behavioral tests. For ACh infusion in the hypothalamus, mice were anesthetized with Zoletil and Rompun, a guide cannula was placed 1.8 mm AP, 0.8 mm L and 4.7 mm Depth from the bregma, and it was fixed with quick-setting cement. After 7 days, mice were infused via cannula with ACh (A6625, Sigma-Aldrich) 100 pg/ml in 3 μL PBS. For INF-γ infusion in the PFC, the guide cannula was placed +2.2 mm AP, ±1.5 L and −1 mm Depth from bregma, and XMG1.2 was infused 3.6 μg/ml in 2 μl PBS. Specifically, a needle was inserted into the cannula and connected to the Hamilton syringe, and the administration was made by an infusion pump at a constant speed of 0.5 μL/min. The day after the infusion, animals were subjected to behavioral tests.

### Behavior

Experimental groups were blinded and randomly assigned before the start of behavioral experiments and remained blinded until all data were collected. Most of our behavior studies were performed on male mice, and our key findings were also tested in female mice (Supplementary Fig. S3a, b). Unless stated otherwise, mice were tested at 10–12 weeks of age. Sample sizes were chosen on the basis of a power analysis using estimates from previously published experiments. For cohorts tested with multiple behavioral assays, the elevated plus maze was performed first and then followed by the open-field before any other test, from least to most invasive. The time between the different behavioral tests was 24 h. Before all experiments, mice were transported to the behavior room and left at least 30 min to habituate.

#### Locomotor activity test

Distance moved (m) was performed in the apparatus comprised eight gray opaque Plexiglas chambers divided into two compartments (20 × 10 cm), with removable floors, placed inside a sound-attenuated room. Mice were placed in the activity chambers for 60 min.

#### Elevated plus maze

The elevated Plus Maze (EPM) apparatus comprised a central section (5 × 5 cm); 2 opposing open arms (15 × 5 cm), and 2 opposing closed arms (15 × 5 × 15 cm). The percentage of time spent in the open arms [(time in open/open closed) × 100], the percentage of entries in the open arms [(open entries/open closed) × 100], and the distance traveled in the apparatus were recorded for 5 min and automatically scored with “EthoVision” (Noldus, The Netherlands) software.

#### Open-field test

This test is used to assay general locomotor activity levels, anxiety-like, and willingness to explore. Individual mice were placed in the corner of an enclosed platform (40 cm × 40 cm × 30 cm). The total distance traveled, movement duration and time spent in the center area (20 cm × 20 cm) were recorded for 10 min and automatically scored with ANYMAZE 7.0 (Ugo Basile, Italy) software.

#### Novel object recognition (NOR) test

This test analyzes the non-spatial working memory function. NOR test was conducted in an observation chamber (40 cm × 40 cm × 30 cm) with discriminated objects (A, B and C) identically sized. The NOR test consists of two sessions: the training session and test session. A preceding 10 min of acclimatization to the experimental set-up was carried out to reduce the impact of anxiety-like and stress on the outcomes. The two identical objects (object A and B) were symmetrically fixed to the floor of the box. The training session was carried out by placing each mouse in the middle of the two objects, and each mouse was allowed to explore the objects for 10 min. After the training, the animals were immediately returned to their home cages, and the observation chamber and objects were cleaned with 70% ethanol to avoid innate odorant cues from previous animal during the training phase. An animal was considered to be exploring the object when its head was facing the object i.e., the distance between the head and object is an approximately 1 cm or less, or was touching or sniffing the object. Test sessions were carried out 1 h and 24 h after the habituation session. In the test session, object B used during training session was replaced with a new object (C, novel to the mice). Mice were placed back to the box and allowed to freely explore objects A and C for 5 min, and the all the behavioral parameter are calculated by software ANY MAZE 7.0.

### Measurement of IFN-γ, ACh and DA by ELISA

The indicated brain regions of male mice were disrupted with a homogenizer and analyzed for IFN-γ, ACh and DA content using a sandwich ELISA, following the manufacturer’s instructions (IFN-γ ELISA kit (KAC1231) was from Life technologies Invitrogen (Carlsbad, CA, USA); ACh ELISA kit (#E4453-100) was from BioVision (Milpitas, CA, USA); DA ELISA kit (KA1887) was from Abnova (Walnut, CA, USA). Briefly, 96-well ELISA microplates were coated with specific monoclonal Ab. Samples or standard were added at the appropriate dilution and incubated for 2 h at room temperature. After careful washing, biotinylated goat anti- IFN-γ, ACh or DA were added to each well; horseradish-peroxidase was used as secondary Ab and optical density was read at 450 nm.

In order to quantify ACh released by NK cell in vitro, purified murine and human NK cells were activated O/N in IL-15 (20 ng/ml) and then co-incubated for 48 h with PMA/ionomycin (1:100), IL-15 (50 ng/ml), IL-2 (50 ng/ml), or with primary microglia at ratio 1:1. Then medium was collected and analyzed for ACh ELISA kit (#E4453-100).

### Microdialysis

Mice, anaesthetized with Zoletil and Rompun, were mounted on a stereotaxic frame (David Kopf Instruments) and implanted unilaterally with microdialysis probes 24–36 h before experiments. The concentric dialysis probes (AN69 fibers, Hospal Dasco) were implanted vertically at the level of the hippocampus (AP − 3.0, ML ± 2.7 from bregma). The probe length was 5 mm (3 mm membrane). Each probe was fixed and the skin was sutured and mice were returned to their home cages until the day of experiment. Membranes were tested for in vitro recovery before surgery. On the day of the experiment each animal was placed in a circular cage containing microdialysis equipment: the microdialysis probe was connected to a CMA/100 pump (Carnegie Medicine) through PE-20 tubing and an ultra-low torque multichannel power-assisted swivel (Model MCS5, Instech Laboratories) to allow free movement. Artificial cerebrospinal fluid (aCSF; in mM: NaCl 140; KCl 4; CaCl2 1.2; MgCl2 1) was pumped through the dialysis probe (2.1 μl/ min). Following the start of the dialysis perfusion, mice were left undisturbed for ∼1 h before the collection of six baseline samples to calculate the average basal concentration. Dialysate samples were collected every 20 min for 120 min. Brains were then postfixed in 4% paraformaldehyde, cut in coronal slices (100 μm) and processed for methylene blue staining. The correct positioning of the probes was confirmed under a microscope. Data from animals not showing proper placement were discarded.

Each dialysate sample (20 μl) was analyzed for the DA concentration by UHPLC. Concentrations (pg 20 μl^−1^) were corrected for probe recovery. The UHPLC system consisted of an UltiMate 3000 (Thermo Fisher Scientific S.p.A.) system and a coulometric detector (UltiMate 3000 ECD-3000RS) provided with an analytical cell (6011RS ultra Coulometric Cell). The electrode 1 was set at 100 mV, and the electrode 2 at 250 mV. A C18 column (ACCLRSLC PA2 2.2U2.1 × 100, Thermo Fisher Scientific S.p.A) maintained at 35 °C was used. The flow rate was 0.25 ml/min. A Sentry Guard precolumn (ACCLAIM, V-2 GUARD) was also used. The mobile phase consisted of methanol (7%) in Na phosphate buffer (0.1 M), Na2 EDTA (1.3 mM), and 1-octane sulfonic acid Na salt (0.25 mM), at pH = 3.65. The detection limit of the assay was 0.1 pg. Data were analyzed with one-way repeated measures ANOVAs with treatment (IgG Vs aNK1.1) as between-subject factor and time (20 min blocks, 6 levels) as within-subject factor. The data are reported as average of 6 time point considered.

### Spontaneous inhibitory postsynaptic currents (sIPSCs) and miniature (mIPSCs) recording from L5 in the prefrontal cortex

IgG, aNK1.1- and XMG1.2-treated mice were anesthetized using halothane and decapitated for brain collection. Coronal slices, 350 μm in thickness, were cut in glycerol-based artificial cerebrospinal fluid (ACSF) with a vibratome (Leica VT 1000S) immediately after collection. Slices were placed in a slice incubation chamber at room temperature with oxygenated ACSF and transferred to a recording chamber within 1–6 h after slice preparation. Spontaneous inhibitory postsynaptic currents (sIPSCs) were recorded from L5 pyramidal neurons in the prefrontal cortex at 22–25 °C using a Multiclamp 700B amplifier (Axon Instruments, Foster City, CA, USA), −70 mV holding potential, in the presence of 6-cyano-7-nitroquinoxaline-2,3-dione (CNQX, 20 µM) and D-(-)−2-Amino-5-phosphonopentanoic acid (AP5, 40 µM) to block AMPA and kainate receptors, respectively. Miniature inhibitory postsynaptic currents (mIPSCs) were recorded in the same conditions, in presence of tetrodotoxin (TTX, 1 μM). ACSF had the following composition: 125 mM NaCl, 2.5 mM KCl, 2 mM CaCl2, 1.25 mM NaH2PO4, 1 mM MgCl2, 26 mM NaHCO3, 10 mM glucose (pH 7.35). Glycerol-based ACSF solution contained 250 mM glycerol, 2.5 mM KCl, 2.4 mM CaCl2, 1.2 mM MgCl2, 1.2 mM NaH2PO4, 26 mM NaHCO3, 11 mM glucose (pH 7.35). Patch pipettes were filled with 140 mM KCl, 10 mM HEPES, 5 mM 1,2-bis(2-aminophenoxy)ethane-N,N,N′,N′-tetraacetic acid (BAPTA), 2 mM MgCl2, and 2 mM Mg-ATP (pH 7.35, with KOH). sIPSCs kinetic analysis was performed using Clampfit 11 software (Molecular Devices) with a template-based detection algorithm. The rise time (rt) was estimated as the time taken by the current to increase from 10% to 90% of the peak and the decay time (dt) as the time taken by the current to decrease from 90% to 10%. The inhibitory charge of a single synaptic event (Q) was measured as the time integral of the spontaneous GABAA-mediated synaptic currents. For statistical comparison One-way ANOVA was performed and the statistical significance was set at *p* < 0.05; statistical significance of cumulative distributions of amplitudes and interevent-intervals was assessed using the Kolmogorov–Smirnov test with Clampfit 11 software.

### Spontaneous excitatory and inhibitory postsynaptic currents recording from dopaminergic neurons in the VTA

Mice were anaesthetized with halothane and perfused transcardially with cold (0.5–4 C), oxygenated (95% O_2_, 5% CO_2_) ‘slicing’ solution, containing (mM): NMDG 92, KCl 2.5, NaH_2_PO_4_ 1.2, NaHCO_3_ 30, HEPES 20, Glucose 25, Na-ascorbate 5, Thiourea 2, Na-pyruvate 3, MgSO_4_ 10, CaCl_2_ 0.5 (pH 7.4 with HCl, 295–310 mOsm). At perfusion completion, mice were decapitated, and brains rapidly removed and dissected. Horizontal slices containing the VTA (250 μm-thick) were cut using a vibratome (HM 650 V; Microm International GmbH, Walldorf, Germany) in chilled, oxygenated slicing solution. After cutting, brain slices were transferred into a holding chamber containing the same solution and incubated at 34 °C for 25 min (according to mouse age) while slowly increasing NaCl to a final concentration of approx. 90 mM. Finally, slices were transferred into a recovery chamber with ‘holding’ solution, containing (mM): NaCl 92, KCl 2.5, NaH_2_PO_4_ 1.2, NaHCO_3_ 30, HEPES 20, Glucose 25, Na-ascorbate 5, Thiourea 2, Na-pyruvate 3, MgSO_4_ 2, CaCl_2_ 2 (pH 7.3-7.4 with NaOH, 300–310 mOsm) where they were kept at room temperature until use.

For patch-clamp recordings slices were transferred to a submerged chamber and perfused with oxygenated artificial Cerebro-Spinal Fluid (aCSF; 3–4 ml/min) containing (in mM): NaCl 125, KCl 3, NaHCO_3_ 26, NaHPO_4_ 1.25, Glucose 25, MgCl_2_ 1, CaCl_2_ 2 (pH ~7.3; 300–310 mOsm). Putative dopaminergic neurons of the ‘lateral’ VTA were visualized using the IR-DIC optics of an upright Leica DMLSF microscope (40X) and voltage-clamp recordings were obtained using a MultiClamp 700B amplifier and the pClamp9 suite (Molecular Devices). Neurons were selected for recording according to the cell location and to the presence of low-frequency, regular firing of action potentials in cell-attached configuration. These criteria were validated on PFA (4% o.n.) post-fixed slices processed for double IF staining of DAT^CRE-yfp^ dopaminergic neurons and biocytin/streptavidin-Alexa 555. All experiments were performed at near-physiological temperature (32–34 °C). In voltage-clamp recordings, patch-pipettes were pulled (PC-10, Narishige) from thick-walled borosilicate glass (1.5 mm outer diameter and 0.86 mm inner diameter; GC150F-7.5; Harvard Apparatus), coated with Sylgard resin (Dow-Corning 184) and fire-polished (MF-830, Narishige) to a tip resistance of 3–7 MΩ when filled with an ‘intracellular’ pipette solution containing (in mM): Cs-methanesulfonate 115, CsCl 10, HEPES 10, Mg_2_ATP 4, Na_2_GTP 0.3, CaCl_2_ 0.45, Sodium-Phosphocreatine 10, QX314-Cl 5, EGTA 1, Biocytin 0.2–0.4% (pH adjusted to 7.3 with CsOH; 280–290 mOsm). To improve whole-cell voltage-clamp, series resistance (*R*_*s*_) was compensated (range 25–50%; average *R*_*s*_ value after compensation 12.8 ± 0.8 MΩ, *n* = 17) and monitored along the experiment; recordings with compRs >20 MΩ were discarded.

sEPSCs or sIPSCs were recorded consecutively while holding the membrane potential of patched neurons at, respectively, –70 or 0 mV. The estimated reversal potential (E_rev_) for GABAergic sIPSCs was approx. –66 mV and for glutamatergic sEPSCs was approx. 0 mV, thus no blockers were necessary to isolate glutamatergic or GABAergic currents at the chosen holding potentials. All synaptic currents were filtered at *f*_*c*_ 4 kHz and sampled at 20 kHz (amplifier in-built 8-pole low-pass Bessel filter). Spontaneous excitatory and inhibitory synaptic events were detected non-automatically using an event template (100–200 events-based) and analyzed (dwell times shorter than 1 ms were skipped; Clampfit 11 software). Three different analysis windows (at least 60-sec long each) per cell were scanned and genuine synaptic event collected (average number of events per cell: 574 ± 118 and 3703 ± 384, for sEPSCs and sIPSCs, respectively; range: 2150–6726 and 75–1411, respectively). From binned events, average peak amplitude, instantaneous frequency (inter-event interval) and total charge transfer (integral of synaptic event) were estimated for each cell.

### Fiber photometry and optogenetic VTA stimulation

DAT^CRE-yfp^ mice were anesthetized for stereotaxic surgeries. For optogenetic stimulation, the virus (rAAV5-EF1α-DIO-hChR2-eYFP 500 nl) was injected into the VTA (AP: −3.4; ML: −0.5; DV: −4.2 from the dura) unilaterally, through a stainless steel 33-gauge internal cannula attached to a 10 μl Hamilton syringe, at a rate of 0.5 μl min (1.5 μl total volume). After infusion, the cannula was kept at the injection site for 3 min and then slowly withdrawn. Subsequentially, mice also received surgical implantation of a monofiberoptic cannula (200 μm, 0.22NA, Ugo Basile), above the VTA. We used surgical sutures to close the skin, and the mouse was kept in a warm environment until resuming normal activity. Mice were allowed to recover for 3 weeks, and then tested for behavioral analysis. Each cable was flexible so that mice could freely move about their cages. We found that 95.1 ± 2.6% of eYFP cells in the VTA co-expressed tyrosine hydroxylase (TH) (Supplementary Fig. S6a). In detail, mice were connected to fiber optic patch cords (Ugo Basile) using ceramic sleeves. Optogenetic stimulations (15 ms pulses at 1–25 Hz) were generated by a waveform generator (Intelligent optogenetics system, Ugo Basile, Italy) that triggered blue-light lasers (473 nm), for 3 s with 5-s cue light illumination and the house light off. The output power of the laser was adjusted to 20 mW transmittance into the brain.

### Immunostaining

Mice were anesthetized and intracardially perfused with PBS and then 4% formaldehyde; brains were then isolated, fixed in 4% formaldehyde and snap frozen. Cryostat sections (20 μm) were washed in PBS, blocked (3% goat serum in 0.3% Triton X-100) for 1 h, at RT, and incubated overnight at 4 °C with specific antibodies diluted in PBS containing 1% goat serum and 0.1% Triton X-100. The sections were incubated with the following primary Abs: anti-c-fos 1:500 (Abcam Cat# ab208942; RRID:AB_2747772); anti-Orx 1:50 (Cell Signaling Technology Cat# 16743, RRID:AB_2798770); anti-tyrosine hydroxylase 1:150 (Millipore Cat# AB9983, RRID:AB_1587573). After several washes, sections were stained with the fluorophore-conjugated antibody and Hoechst (Cell Signaling Technology Cat# 4082, RRID:AB_10626776) for nuclei visualization and analyzed using a fluorescence microscope. For co-immunofluorescence, the secondary antibody was subsequently used. For all the antibodies staining, coronal sections were first boiled for 20 min in citrate buffer (pH 6.0) at 95–100 °C. Images were digitized using a CoolSNAP camera (Photometrics,Tucson, USA) coupled to an ECLIPSE Ti-S microscope (Nikon, Tokyo, Japan). Fos-immunopositive neurons were counted manually using MetaMorph 7.6.5.0 image analysis software (Molecular Device, San Jose, USA). c-Fos^+^ neurons were determined only when nuclear expression of c-Fos co-localized with Hoechst 33342 staining.

### Statistical analysis

Data are shown as the mean ± S.E.M. All the measurements were taken from distinct samples. Statistical significance was assessed by Student’s *t* test, one-way ANOVA or two-way ANOVA for parametrical data, as indicated; Holm–Sidak test was used as a post-hoc test; Mann–Whitney Rank test and Kruskal–Wallis for non-parametrical data, followed by Dunn’s or Tukey’s post-hoc tests. For multiple comparisons, multiplicity adjusted p-values are indicated in the corresponding figures. Statistical analyses comprising calculation of degrees of freedom were done using Sigma Plot 11.0, GraphPad Prism 9.0, Imaris 8 and Origin 7. For each experiment, the sample size (*n*) was chosen considering the following relation: n ≥ 2sigma (Zalpha/D)2, where sigma is substituted by an estimate of variance (s2); alpha is at 0.05 (and Zalpha = −2) and D is the difference among treatments. Criteria of animal exclusion/inclusion were pre-established; animals considered for the analysis were selected for age. At weaning, pups from different colonies were mixed and mice were randomly treated. The investigators performing the different analyses always received the samples from a third laboratory member, who was not involved in that specific experiment, to ensure blinding to the group allocation.

### Reporting summary

Further information on research design is available in the Nature Portfolio Reporting Summary linked to this article.

## Supplementary information

Supplementary Information

Supplementary Table 1

Reporting Summary

## Data Availability

The accession number for the single-cell sequencing data described in this paper is GSE212089. The source data are provided as a Source Data file. Datasets generated during and/or analyzed in this study are available from the corresponding author upon request. Source data are provided with this paper.

## Source data

Source Data
