# Supplementary material for: Natural killer cells and innate lymphoid cells 1 tune anxiety-like behavior and memory in mice via interferon-γ and acetylcholine
Source: Nat Commun. 2023 May 29;14:3103. doi: 10.1038/s41467-023-38899-3 (PMC10227025; doi:10.1038/s41467-023-38899-3)
Supplement: Supplementary file 1 — Supplementary Information [file 41467_2023_38899_MOESM1_ESM.pdf]

Supplementary information for the manuscript:

Natural killer cells and innate lymphoid cells 1 tune anxiety and memory via interferon-γ and acetylcholine

Garofalo et al.

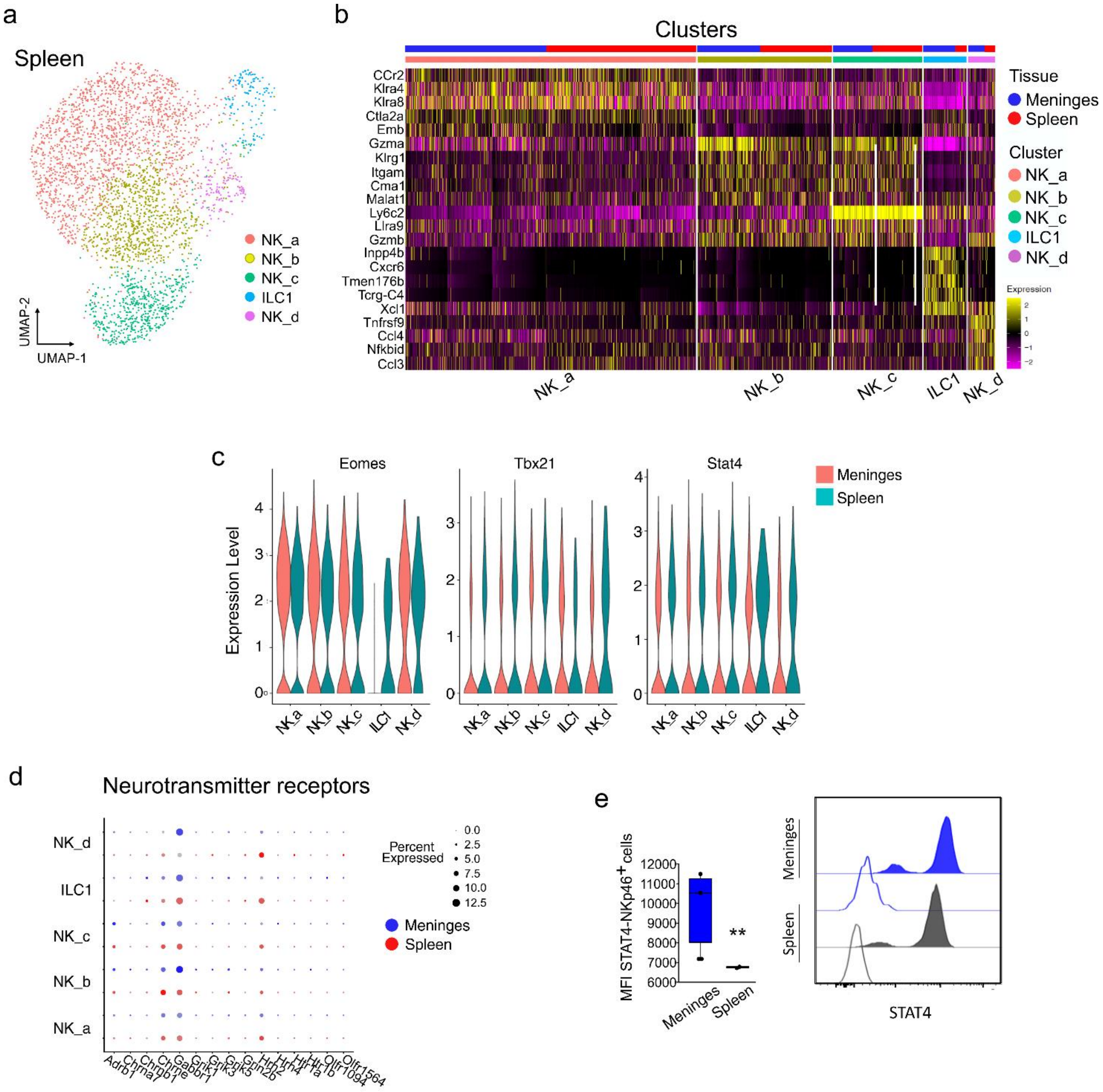

Supplementary Fig. 1: a) UMAP representation of $CD3^-$/NK1.1$^+$ cells deriving from spleen (3387 cells). Profiles colored are from clusters identified subsets of NK cells and ILC1. **b)** Gene expression heatmap of the gene markers per cluster. **c)** Violin plots represent the distribution of indicated genes grouped by clusters and divided for tissue. **d)** DotPlot represent the expression levels of ILCs archetypal genes within the identified NK cells and ILC1 clusters in meninges and spleen. **e)** Left: box plot showing quantification of STAT4 expression by MFI ± SD in the $CD3^-CD19^-NKp46^+$ cell population obtained from the meninges and spleen (n = 4 **p<0.001 two-tailed Student's t-test). Right: representative histogram plots of meningeal and splenic STAT4 expression by spleen (grey) and meningeal (blue) $CD3^-CD19^-NKp46^+$ cells. Empty histograms show isotype control, while filled histograms show stained samples. For boxplots, the center line, boxes and whiskers represent the median, inner quartiles, and rest of the data distribution, respectively.

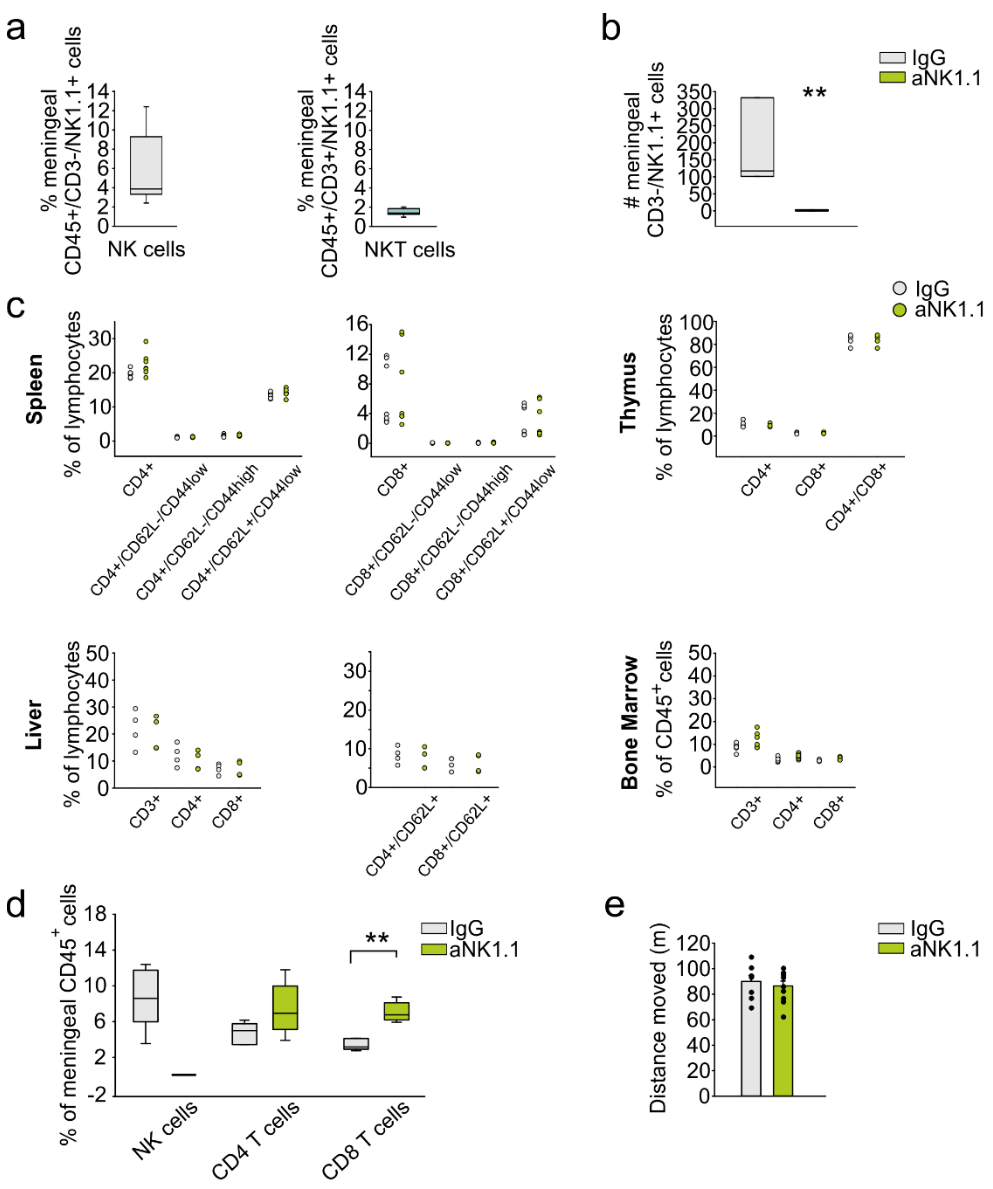

Supplementary Fig. 2: a) FACS analysis of NK cells and NKT cells frequency in the meninges of C57BL/6 mice (n = 5). **b)** FACS analysis of CD3$^-$/NK1.1$^+$ cell number in the meninges of mice treated with IgG or aNK1.1 (i.p.) as indicated (n = 3 mice per conditions, **p<0.001 two-tailed Student's t-test). **c)** FACS analysis of the frequency and distribution of T cell subsets (CD4$^+$ and CD8$^+$ and naïve CD62$^+$CD44$^-$ and memory CD62$^+$CD44^{high} cells among CD4$^+$ and CD8$^+$ cells) in spleen, liver, bone marrow, and thymus after 4 weeks of treatment with IgG or aNK1.1 (n = 7 mice per condition). **d)** FACS analysis of the frequency of NK cells and CD4 and CD8 T lymphocytes collected from the meninges of IgG- and aNK1.1-treated mice (n = 10 mice per condition, *p<0.05 **p<0.01 one-way ANOVA). For boxplots (**a,b,d**) the center line, boxes and whiskers represent the median, inner quartiles, and rest of the data distribution, respectively. **e)** Total locomotor activity measured as the total distance traveled over the open-field test in 1 h (n = 10 IgG- and aNK1.1treated mice). Data are expressed as mean ± SEM.

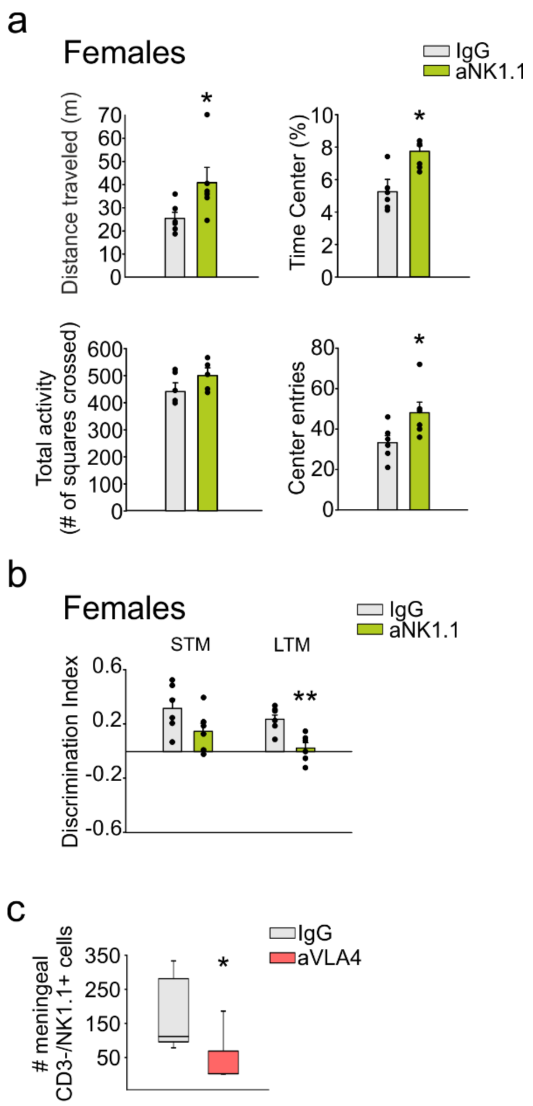

Supplementary Fig. 3: a) Open-field test (10 min) in IgG- and aNK1.1-treated female mice (n = 6 mice per condition), showing total distance travelled (*p=0.016), percent time spent in the center (*p=0.015), center episodes (*p=0.040), and the total activity (p=0.219; two-tailed Student's t-test). Data are expressed as mean ± SEM. **b)** Object novelty discrimination after 1h (STM) and 24h (LTM) in female mice treated with IgG and aNK1.1 (n = 6 mice per condition, **p=0.003 IgG vs aNK1.1; one-way ANOVA). Data are expressed as mean ± SEM. **c)** FACS analysis of $CD3^-/NK1.1^+$ cell number in the meninges of mice treated with IgG or aVLA4 as indicated (n = 6 mice per conditions, *p=0.011 two-tailed Student's t-test). For boxplots, the center line, boxes and whiskers represent the median, inner quartiles, and rest of the data distribution, respectively.

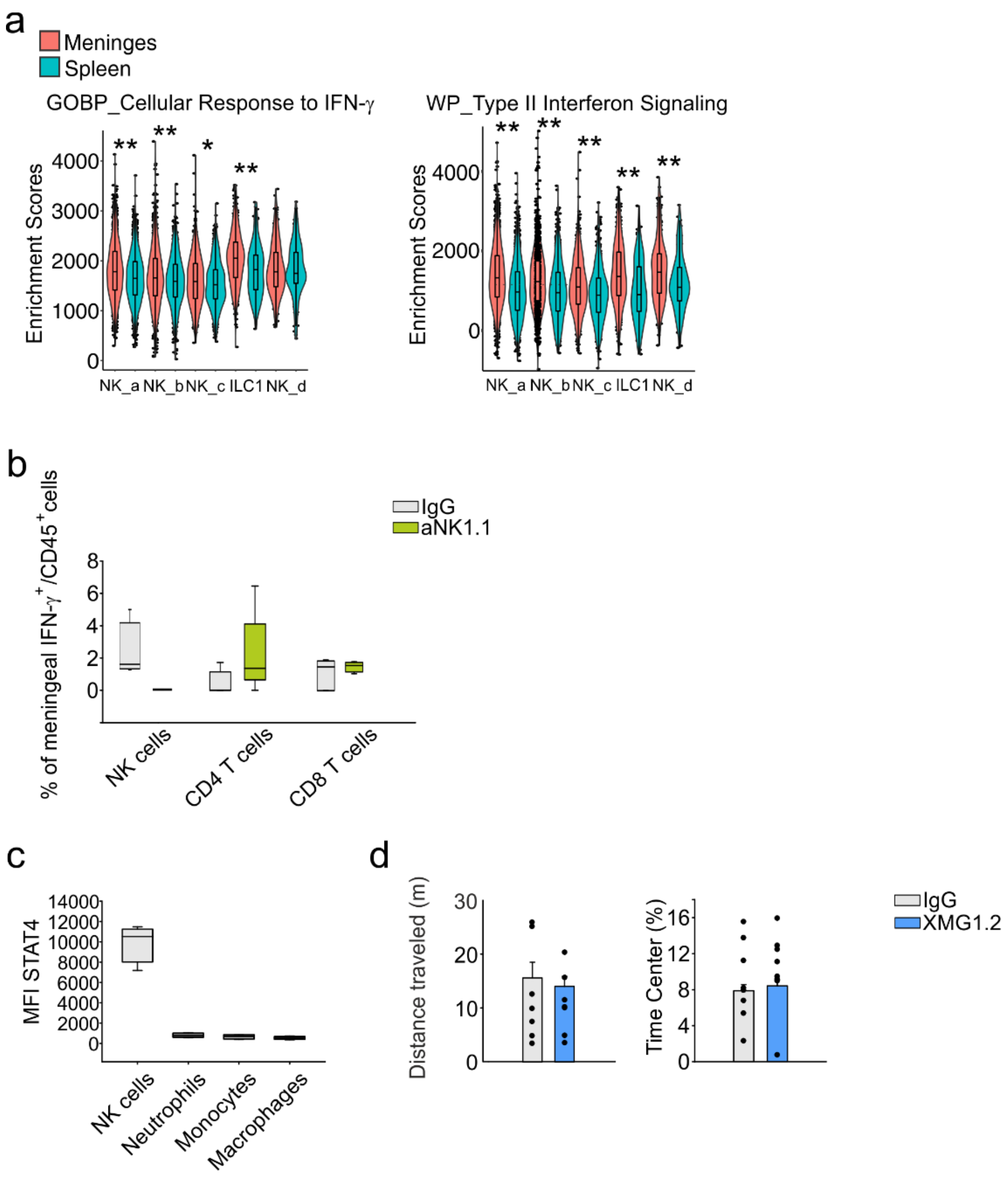

Supplementary Fig. 4: a) Violin plot of the distribution of genes related to IFN-γ cellular response and signaling in different clusters and tissue (n=50 mice *p<0.05 **p<0.01 Kruskal-Wallis post-hoc Dunn test). **b)** FACS analysis of the frequency of IFN-γ^+ CD4 and CD8 T lymphocytes collected from the meninges of IgG- and aNK1.1-treated mice (n = 10 mice per condition). **c)** FACS analysis of STAT4 MFI in different cell population obtained from the meninges of C57BL/6 mice (n = 4). For boxplots (**a,b,c**) the center line, boxes and whiskers represent the median, inner quartiles, and rest of the data distribution, respectively. **d)** Open-field test (10 min) results for IgG- and XMG1.2-treated mice (n = 8 mice per condition), showing total distance travelled and percent time spent in the center. Data are expressed as mean ± SEM.

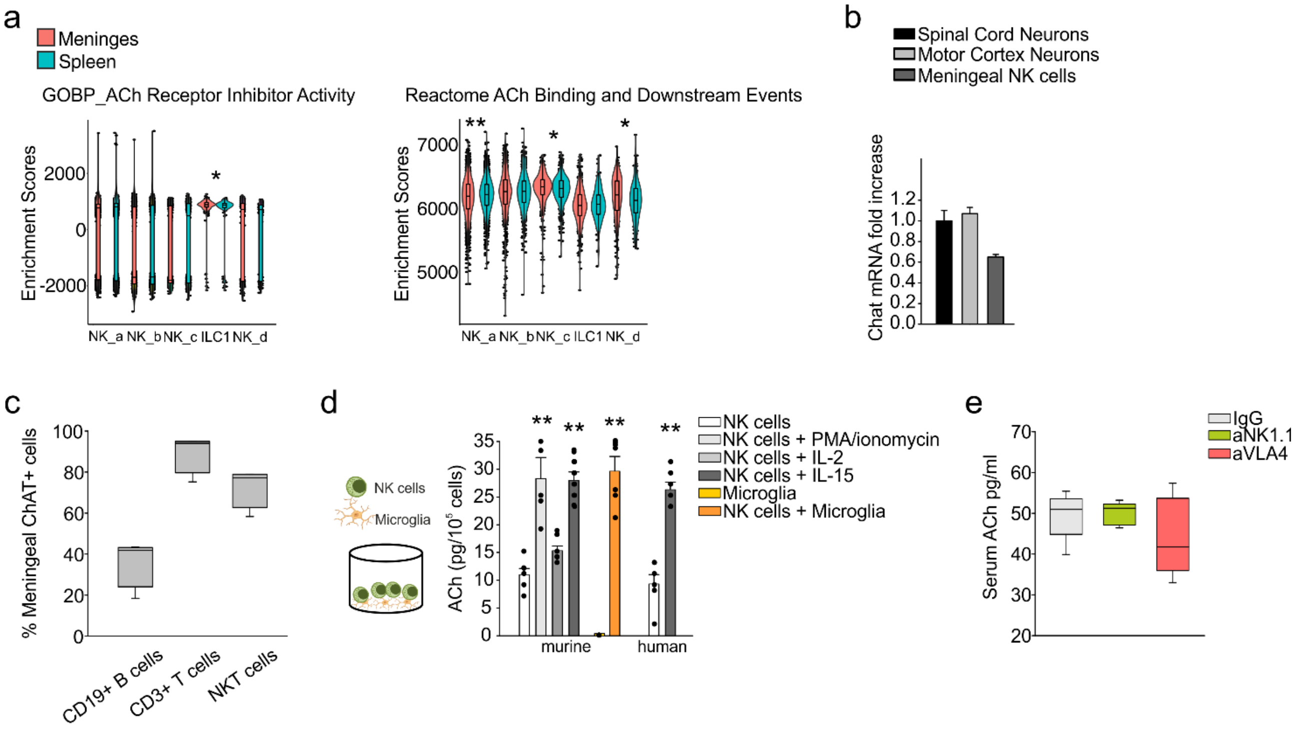

Supplementary Fig. 5: a) Violin plot of ACh inhibitory activity and downstream events gene in different clusters and tissue (n=50 mice *p<0.05 **p<0.01 Kruskal-Wallis post-hoc Dunn test). **b)** qPCR analysis of *Chat* mRNA in neurons collected from spinal cord and motor cortex, and from meningeal NK cells of 2-month-old C57BL/6 mice (n = 5). Data are expressed as mean ± SEM. **c)** FACS analysis of frequency of $ChAT^{+}$ cells in the CD19, CD3 and NKT cells obtained from the meninges (n = 8). **d)** Elisa quantification of ACh released by cultured human or murine NK cells stimulated with phorbol 12-myristate 13-acetate (PMA)/ionomycin (PI), IL-2, or IL-15 or in co-culture with primary murine microglia for 48 h (n=6 **p<0.001 two-tailed Student's t-test). **e)** Elisa quantification of ACh in the serum of IgG-, aNK1.1-, and aVLA4-treated mice (n = 5 mice per condition). For boxplots (**a,c,e**) the center line, boxes and whiskers represent the median, inner quartiles, and rest of the data distribution, respectively.

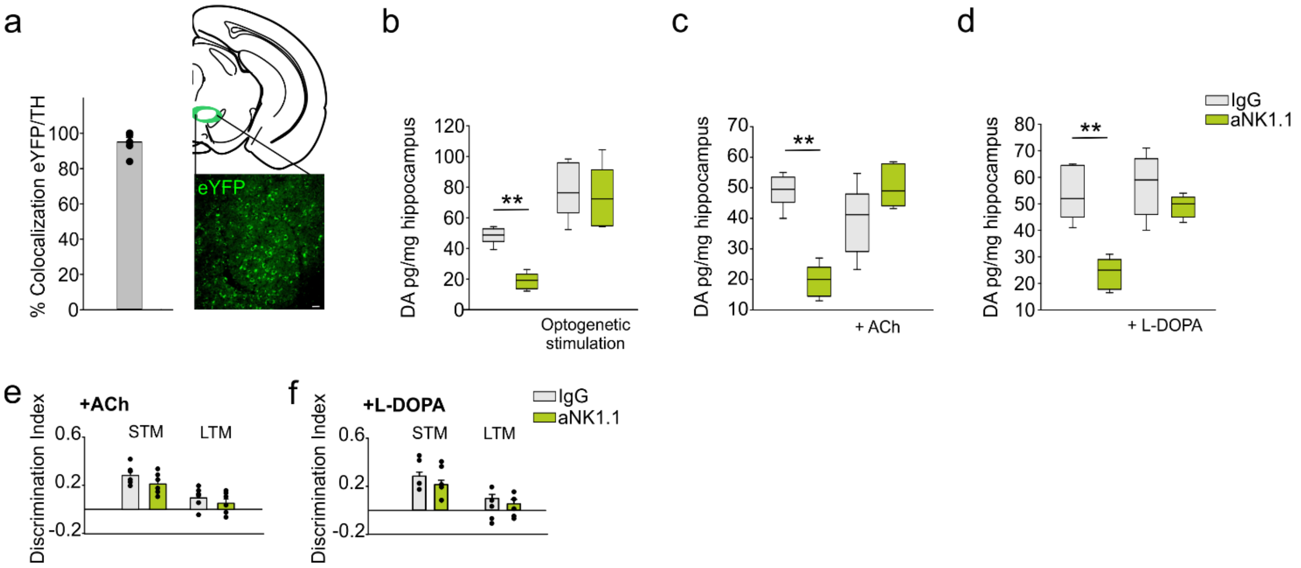

Supplementary Fig. 6: a) Colocalization of eYFP and TH staining in VTA of DAT$^{CRE\text{-}yfp}$ mice three weeks after rAAV injection (n = 6). Scale bar: 50 µm. **b-d)** Expression of DA in the hippocampus of mice treated with IgG or aNK1.1: after optogenetic stimulation (n = 6 mice per condition, **p<0.001 two-tailed Student's t-test, **b**); after intracerebral infusion of ACh (n = 5 mice per condition, **p<0.001 two-tailed Student's t-test, **c**); and after i.p. treatment with L-DOPA (n = 5 mice per condition, **p<0.001 two-tailed Student's t-test, **d**). For boxplots (**b,c,d**), the center line, boxes and whiskers represent the median, inner quartiles, and rest of the data distribution, respectively. **e,f**) Discrimination index in NOR test after 1h (STM) and 24h (LTM) in IgG and aNK1.1 mice treated with intracerebral infusion of saline or ACh (n = 6 mice per condition; **e**), and i.p. injected with saline or L-DOPA (n = 5 mice per condition; **f**), as indicated. Data are expressed as mean ± SEM.

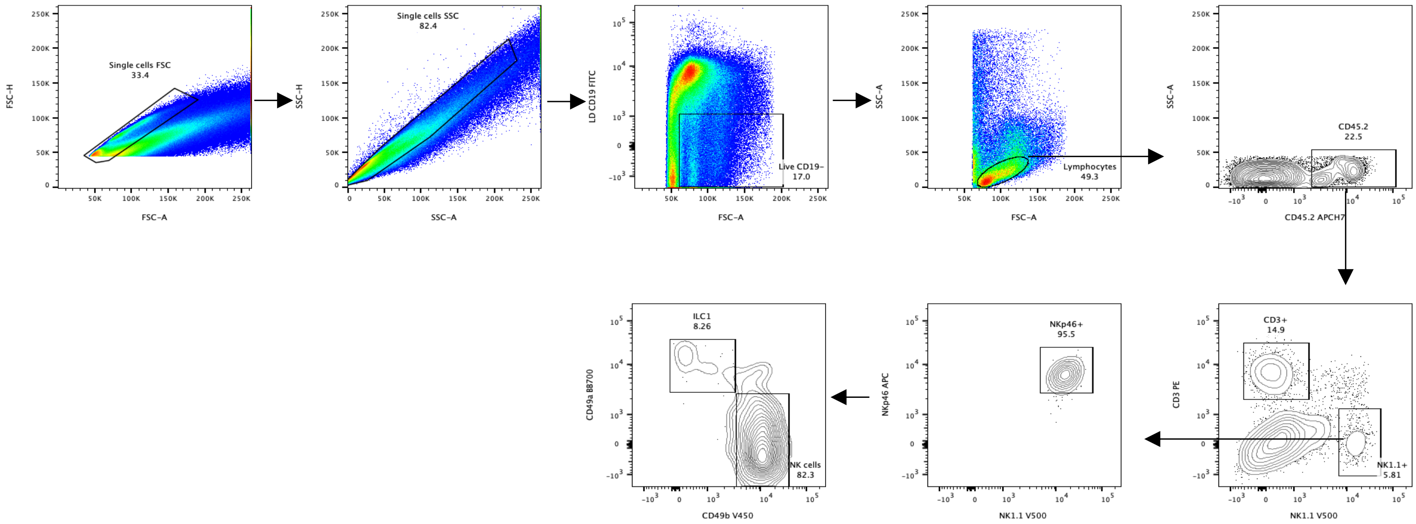

Supplementary Fig. 7: Gating strategies. Hematopoietic cells were obtained from mouse meninges upon enzymatic digestion and mechanical disruption on a 70 µm cell strainer and were labeled with fixable/viability dye to exclude death cells and with anti-CD45, -CD3 and -NK1.1. ILC group 1 were identified as $CD3^-$ /$NK1.1^+$ cells among $CD45^+$ cells. NK cell and ILC1 were identified as $CD49b^+$ and $CD49a^+$ cells respectively, among $NKp46^+$ cells.

A

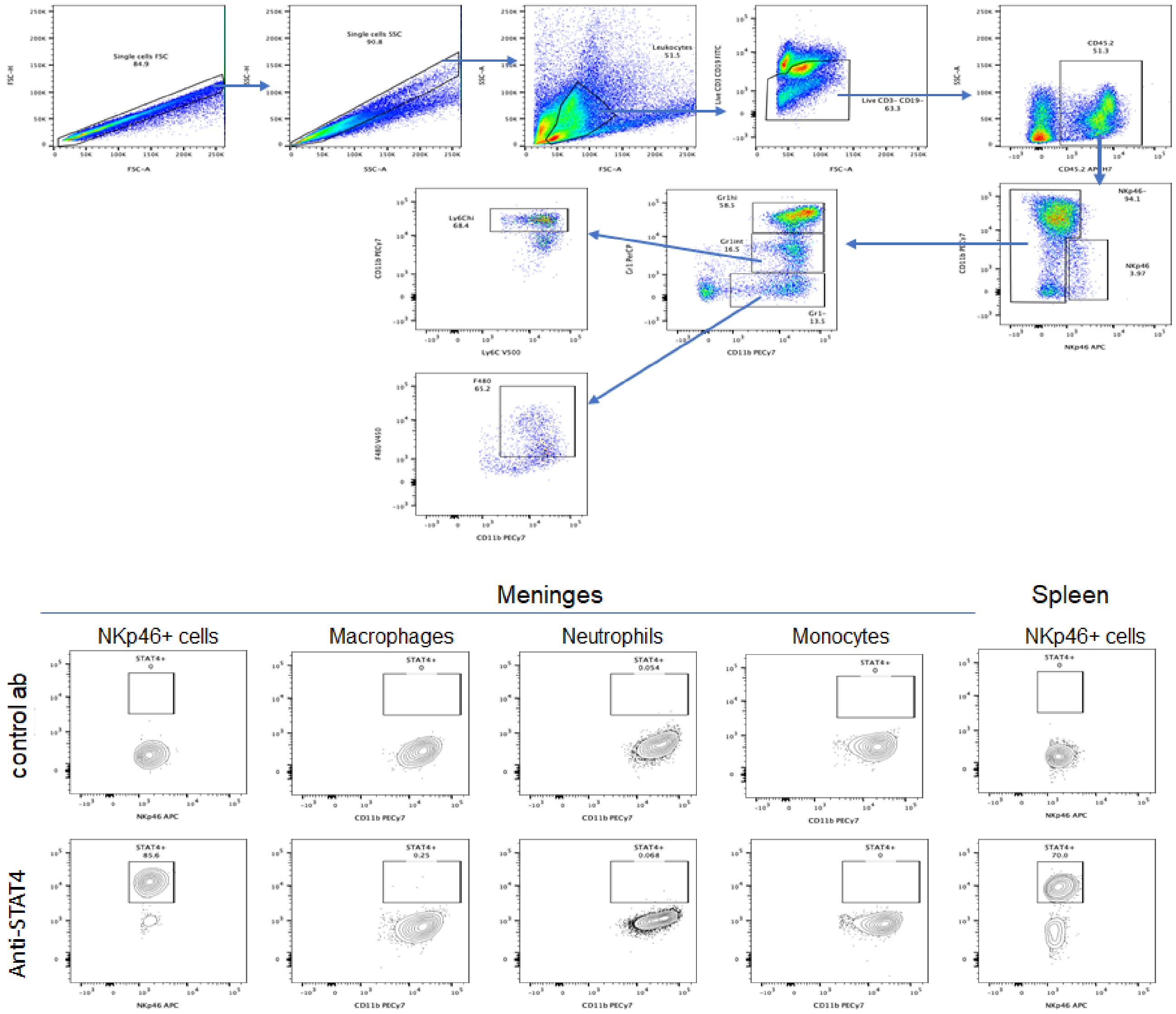

B

Supplementary Fig. 8: A) Representative gating strategies. Hematopoietic cells were obtained from mouse meninges upon enzymatic digestion and mechanical disruption on a 70 µm cell strainer and were labeled with fixable/viability dye to exclude death cells and with anti-CD45, -CD3, -NKp46, -CD11b, -Gr1, -Ly6c, and -F4/80. Cells were permeabilized and stained with anti-STAT4 or control antibodies. NK/ILC1 were identified as $CD3^{-}CD19^{-}NKp46^{+}$, monocytes as $CD11b^{+}Gr1^{int}Ly6c^{high}$, macrophages as $CD11b^{+}Gr1^{neg}F4/80^{+}$, neutrophils as $CD11b^{+}Gr1^{high}$ cells among $CD45^{+}$ cells. **B)** Dot plot analysis of STAT4 expression in hematopoietic cell populations in meninges and spleen.

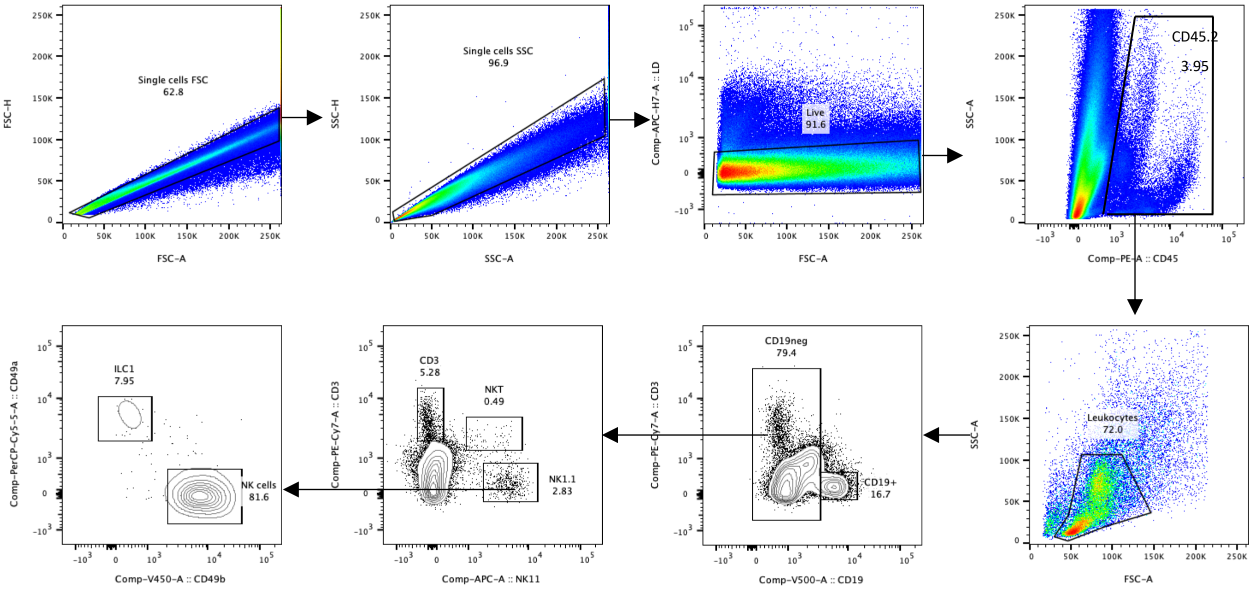

Supplementary Fig. 9: Gating strategies. Hematopoietic cells were obtained from mouse meninges upon enzymatic digestion and mechanical disruption on a 70 µm cell strainer and were labeled with fixable/viability dye to exclude death cells and with anti-CD45, -CD3, -CD19 and -NK1.1.
