# Supplementary material for: Natural killer cells and innate lymphoid cells 1 tune anxiety-like behavior and memory in mice via interferon-γ and acetylcholine
Source: Nat Commun. 2023 May 29;14:3103. doi: 10.1038/s41467-023-38899-3 (PMC10227025; doi:10.1038/s41467-023-38899-3)
Supplement: Supplementary file 3 — Reporting Summary [file 41467_2023_38899_MOESM3_ESM.pdf]

nature portfolio

Corresponding author(s): Cristina Limatola and Stefano Garofalo

Last updated by author(s): 02/05/2023

Reporting Summary

Nature Portfolio wishes to improve the reproducibility of the work that we publish. This form provides structure for consistency and transparency in reporting. For further information on Nature Portfolio policies, see our Editorial Policies and the Editorial Policy Checklist.

Statistics

For all statistical analyses, confirm that the following items are present in the figure legend, table legend, main text, or Methods section.

n/a	Confirmed	
☐	☒	The exact sample size (*n*) for each experimental group/condition, given as a discrete number and unit of measurement
☐	☒	A statement on whether measurements were taken from distinct samples or whether the same sample was measured repeatedly
☐	☒	The statistical test(s) used AND whether they are one- or two-sided<br>*Only common tests should be described solely by name; describe more complex techniques in the Methods section.*
☐	☒	A description of all covariates tested
☐	☒	A description of any assumptions or corrections, such as tests of normality and adjustment for multiple comparisons
☐	☒	A full description of the statistical parameters including central tendency (e.g. means) or other basic estimates (e.g. regression coefficient) AND variation (e.g. standard deviation) or associated estimates of uncertainty (e.g. confidence intervals)
☐	☒	For null hypothesis testing, the test statistic (e.g. *F*, *t*, *r*) with confidence intervals, effect sizes, degrees of freedom and *P* value noted<br>*Give P values as exact values whenever suitable.*
☒	☐	For Bayesian analysis, information on the choice of priors and Markov chain Monte Carlo settings
☒	☐	For hierarchical and complex designs, identification of the appropriate level for tests and full reporting of outcomes
☒	☐	Estimates of effect sizes (e.g. Cohen's *d*, Pearson's *r*), indicating how they were calculated

Our web collection on statistics for biologists contains articles on many of the points above.

Software and code

Policy information about availability of computer code

Data collection	FACSAriaII (BD Biosciences)<br>FACSDiva software (BD Biosciences version 6.1.3)<br>FACSCanto II (BD Biosciences)<br>High Sensitivity D5000 ScreenTape<br>Qubit dsDNA HS Kit (ThermoFisher, #Q32854)<br>NovaSeq6000TM sequencer (Illumina)<br>FlowJo 9.3.2<br>UltiMate 3000 (Thermo Fisher Scientific S.p.A.)
Data analysis	Molecular Signature Database (v7.4)<br>ANYMAZE 7.0<br>Clampfit 11<br>Sigma Plot 11.0<br>Imaris 8<br>Origin 7<br>Prism 7<br>GraphPad Prism 9.0<br>SCENIC (v1.2.4)

For manuscripts utilizing custom algorithms or software that are central to the research but not yet described in published literature, software must be made available to editors and reviewers. We strongly encourage code deposition in a community repository (e.g. GitHub). See the Nature Portfolio guidelines for submitting code & software for further information.

Data

Policy information about availability of data

All manuscripts must include a data availability statement. This statement should provide the following information, where applicable:

- Accession codes, unique identifiers, or web links for publicly available datasets
- A description of any restrictions on data availability
- For clinical datasets or third party data, please ensure that the statement adheres to our policy

The accession number for the single-cell sequencing data described in this paper is GSE212089. The source data are provided as a Source Data file. Datasets generated during and/or analyzed in this study are available from the corresponding author upon reasonable request. The data that support the findings of this study are available from the corresponding author upon reasonable request.

Human research participants

Policy information about studies involving human research participants and Sex and Gender in Research.

Reporting on sex and gender	Peripheral blood mononuclear cells (PBMCs) were from both male and females healthy anonymous donors. Being anonymous the donors are randomly distributed.
Population characteristics	Healthy anonymous donors were recruted between 50.26 ± 10.2 years, of different gender.
Recruitment	Healthy donors stratified for age and sex were enrolled, recruited from the Rare Neuromuscular Diseases Centre of Umberto I Hospital in Rome (ref 3314/25.09.14, protocol n. 1186/14).
Ethics oversight	For patients in Rome, from the Ethic committee of Umberto I Hospital in Rome, informed consent was obtained for the use of blood and approval was obtained from the relevant local ethical committees for medical research.

Note that full information on the approval of the study protocol must also be provided in the manuscript.

Field-specific reporting

Please select the one below that is the best fit for your research. If you are not sure, read the appropriate sections before making your selection.

☒ Life sciences ☐ Behavioural & social sciences ☐ Ecological, evolutionary & environmental sciences

For a reference copy of the document with all sections, see nature.com/documents/nr-reporting-summary-flat.pdf

Life sciences study design

All studies must disclose on these points even when the disclosure is negative.

Sample size	The sample size (n) was chosen differently for experiments of animal behavior, marker expression, etc.. on the basis of standard power calculations (with $\alpha = 0.05$ and power of 0.8), performed for similar experiments that were previously published. In general, statistical methods were not used to re-calculate or predetermine sample sizes.
Data exclusions	Animals considered for the analysis were selected for age. We did not exclude animals or data from the study.
Replication	All attemps to replicate the results were successful. Number of reproductions of each experimental finding is stated in each figure legend.
Randomization	For experiments aimed at exploring the role of NK cells/ILC1, or IFNg or neurotransmitters DA and ACh in mouse behavior, animals were randomly chosen among different colonies before the treatments with the differents drugs. Randomization was not relevant for human tissue studies, which were descriptive studies, and for in vitro experiments, in which each experimental group was treated under the same experimental conditions.
Blinding	The investigators performing behavioral test, immunofluorescence, FACS, etc analyses where blinded to group allocation; they always received the samples from a third laboratory member, not directly involved in data analysis.

Reporting for specific materials, systems and methods

We require information from authors about some types of materials, experimental systems and methods used in many studies. Here, indicate whether each material, system or method listed is relevant to your study. If you are not sure if a list item applies to your research, read the appropriate section before selecting a response.

Materials & experimental systems

n/a	Involved in the study
☐	☒ Antibodies
☒	☐ Eukaryotic cell lines
☒	☐ Palaeontology and archaeology
☐	☒ Animals and other organisms
☒	☐ Clinical data
☒	☐ Dual use research of concern

Methods

n/a	Involved in the study
☒	☐ ChIP-seq
☐	☒ Flow cytometry
☒	☐ MRI-based neuroimaging

Antibodies

Antibodies used

Antibodies used for immunofluorescence studies include: rabbit anti-NKp46 (M20) #sc-18161 (1:50 Santa Cruz); anti-c-fos 1:500 (Abcam Cat# ab208942; RRID:AB_2747772); anti-Orx 1:50 (Cell Signaling Technology Cat# 16743, RRID:AB_2798770); anti-tyrosine hydroxylase 1:150 (Millipore Cat# AB9983, RRID:AB_1587573).
Antibodies used for in vivo depletion include: anti-NK1.1 (0.2 mg Cat# BE0036), anti Rat anti–IFN-γ monoclonal antibody (clone: XMG1.2)(0.2 mg), all from Bioxcell. Anti-VLA-4 (CD49d / Natalizumab) (#BE0071, RRID:AB_1107657) was from Bioxcell (West Lebanon, USA).

Validation

Antibodies used were commercialy available and all the antibodies were validated by manufacturers, with related data shown on the manufacturer's website.
rabbit anti-NKp46 (M20) #sc-18161: https://www.scbt.com/it/p/nkp46-antibody-9e2
anti-c-fos 1:500 (Abcam Cat# ab208942; RRID:AB_2747772): https://www.abcam.com/products/primary-antibodies/c-fos-antibody-2h2-ab208942.html
anti-Orx 1:50 (Cell Signaling Technology Cat# 16743, RRID:AB_2798770): https://www.cellsignal.com/products/primary-antibodies/orexin-d6g9t-rabbit-mab/16743
anti-tyrosine hydroxylase 1:150 (Millipore Cat# AB9983, RRID:AB_1587573): https://www.merckmillipore.com/IT/it/product/Anti-Tyrosine-Hydroxylase-Antibody,MM_NF-AB9983
anti-NK1.1 (0.2 mg Cat# BE0036): https://bioxcell.com/invivomab-anti-mouse-nk1-1-be0036
anti Rat anti–IFN-γ monoclonal antibody (clone: XMG1.2)(0.2 mg): https://bioxcell.com/invivomab-anti-mouse-ifng-be0055
Anti-VLA-4 (CD49d / Natalizumab) (#BE0071, RRID:AB_1107657): https://bioxcell.com/invivomab-anti-mouse-human-vla-4-cd49d-be0071

Animals and other research organisms

Policy information about studies involving animals; ARRIVE guidelines recommended for reporting animal research, and Sex and Gender in Research

Laboratory animals

All studies were performed using adult male and female mice (6-12 weeks old mice) as indicated for each experiments. C57BL/6, Rag2-/- (B6.Cg-Rag2tm1.1Cgn/J, RRID:IMSR_JAX:008449), Rag2-/- γc-/- (C;129S4-Rag2tm1.1Flv Il2rgtm1.1Flv/J, RRID:IMSR_JAX:014593), DATCRE (B6.SJL-Slc6a3tm1.1(cre)Bkmn/J, RRID:IMSR_JAX:006660), and Rag2-/- stat4-/- mice were obtained from Jackson Laboratory (Bar Harbor, ME, USA) and from Charles River (Calco, Italy). Mice were housed in standard breeding cages at a constant temperature (22 ± 1◦C) and relative humidity (50%), with a 12:12 h light:dark cycle (light on 07.00–19.00 h). All experiments were performed during the dark cycle. Food and water were available ad libitum.

Wild animals

The study did not involve wild animals.

Reporting on sex

We used male mice for all the main experiments, except for those presented in Extended data figure 3 showing aNK1.1 effects on female mice.

Field-collected samples

No field-collected samples were used.

Ethics oversight

Experiments described in the present work were approved by the Animal Welfare Body of Sapienza University and the Italian Ministry of Health (authorization n° 775/2020-PR; n° 70/2022; n° 356/2023-PR) in accordance with the guidelines on the ethical use of animals from the European Community Council Directive of September 22, 2010 (2010/63/EU), and from the Italian D.Leg 26/2014.

Note that full information on the approval of the study protocol must also be provided in the manuscript.

Flow Cytometry

Plots

Confirm that:

☒ The axis labels state the marker and fluorochrome used (e.g. CD4-FITC).

☒ The axis scales are clearly visible. Include numbers along axes only for bottom left plot of group (a 'group' is an analysis of identical markers).

☒ All plots are contour plots with outliers or pseudocolor plots.

☒ A numerical value for number of cells or percentage (with statistics) is provided.

Methodology

Sample preparation	Immune cells from mice were enriched by centrifugation on percoll 40%, washed in PBS and immunostained with fluorochrome-conjugated anti-CD3, anti-NK1.1, anti-CD19 and anti-CD45.2 to identify T cells (CD3+NK1.1-) NK cells (NK1.1 +CD3-) and B cells (CD19+). To determine intracellular IFNγ production, cells were maintained in colture for 6 h in the presence of Brefeldine A (10µg/ml), stained with anti-CD56 and -CD3 and subsequently fixed and permeabilized using Cytofix/cytoperm kit (BD Biosciences). After permeabilization, cells were stained with anti-IFN-γ specific mAb and analyzed by FACS.
Instrument	FACSCanto II (BD Biosciences).
Software	FlowJo Version 9.3.2 software (TreeStar).
Cell population abundance	Purity of the sorted cell fractions was confirmed by flow cytometry resulting in a purity of the sorted cells of >98%., as determined by reanalysing by FACS a fraction of sorted cells.
Gating strategy	The Gating strategy is indicated in supplementary materials.

☒ Tick this box to confirm that a figure exemplifying the gating strategy is provided in the Supplementary Information.
